# Hit and go CAS9 delivered through a lentiviral based self-limiting circuit

**DOI:** 10.1038/ncomms15334

**Published:** 2017-05-22

**Authors:** Gianluca Petris, Antonio Casini, Claudia Montagna, Francesca Lorenzin, Davide Prandi, Alessandro Romanel, Jacopo Zasso, Luciano Conti, Francesca Demichelis, Anna Cereseto

**Affiliations:** 1 Centre for Integrative Biology (CIBIO), University of Trento, Laboratory of Molecular Virology, Via Sommarive 9, Trento 38123, Italy; 2 Centre for Integrative Biology (CIBIO), University of Trento, Laboratory of Computational Oncology, Via Sommarive 9, Trento 38123, Italy; 3 Centre for Integrative Biology (CIBIO), Laboratory of Stem Cell Biology, Via Sommarive 9, Trento 38123, Italy

## Abstract

*In vivo* application of the CRISPR-Cas9 technology is still limited by unwanted Cas9 genomic cleavages. Long-term expression of Cas9 increases the number of genomic loci non-specifically cleaved by the nuclease. Here we develop a Self-Limiting Cas9 circuit for Enhanced Safety and specificity (SLiCES) which consists of an expression unit for *Streptococcus pyogenes* Cas9 (SpCas9), a self-targeting sgRNA and a second sgRNA targeting a chosen genomic locus. The self-limiting circuit results in increased genome editing specificity by controlling Cas9 levels. For its *in vivo* utilization, we next integrate SLiCES into a lentiviral delivery system (lentiSLiCES) *via* circuit inhibition to achieve viral particle production. Upon delivery into target cells, the lentiSLiCES circuit switches on to edit the intended genomic locus while simultaneously stepping up its own neutralization through SpCas9 inactivation. By preserving target cells from residual nuclease activity, our hit and go system increases safety margins for genome editing.

## Abstract (web summary)

While CRISPR-Cas9 is a powerful technology, it's *in vivo* application can be limited by unwanted off-target editing events. Here the authors present SLiCES, a self-limiting Cas9 circuit to enhance editing by preventing residual nuclease activity.

Genome editing through the CRISPR-Cas9 technology has tremendous potential for both basic and clinical applications due to its simplicity, target design plasticity and multiplex targeting capacity^1^,^2^,^3^. The main limit in CRISPR-Cas9 utilization is the mutations induced at sites that differ from the intended target which have been detected both *in vitro* as well as in animal models^4^,^5^,^6^,^7^,^8^,^9^,^10^,^11^. This is critical for *in vivo* applications as unwanted alterations could lead to unfavourable clinical outcomes^12^.

An important factor influencing the number of off-target modifications is the amount and persistence of *Streptococcus pyogenes* Cas9 (SpCas9) expression in target cells: high concentrations of the nuclease are reported to increase off-site cleavage, whereas lower amounts of SpCas9 improve the specificity^4^,^5^,^13^,^14^. Moreover, it is likely that any Cas9 protein present after the target locus has been edited has a substantial probability to modify additional sites. Accordingly, transient SpCas9 expression, obtained through direct delivery of recombinant RNA-guided endonucleases (RGENs) complexes into target cells^15^,^16^,^17^ or by using a SpCas9 variant activated by inteins^14^, is sufficient to permanently modify the target genomic locus with decreased off-target activity. However, the delivery of RGENs is highly inefficient and unsuitable for *in vivo* approaches. Although viral vectors are optimal delivery tools, they generate stable expression of the transferred factors which is not necessarily beneficial for CRISPR-Cas9 applications.

Here, we report the development of a self-limiting SpCas9 circuit designed to remove the nuclease from the edited cells. Through an unbiased genome-wide analysis, we demonstrated the complete absence of off-target activity produced by the self-limiting sgRNAs driving the kill-switch. By integrating the Self-Limiting Cas9 circuit for Enhanced Safety and specificity (SLiCES) within a lentiviral delivery vector, we generated an efficient ‘hit and go' SpCas9 delivery system which prevents the accumulation of off-targets resulting in more specific and safer genome editing.

## Results

### The SLiCES circuit

To evaluate the off-target activity produced by long-term expression of SpCas9, we transduced 293-iEGFP cells carrying a single chromosomal copy of enhanced green fluorescent protein (EGFP) with a lentiviral vector expressing SpCas9 together with sgRNAs that can fully (sgGFP-W) or partially (sgGFP-M or sgGFP-MM) anneal to EGFP. The tolerance of SpCas9 for single (sgGFP-M) or double (sgGFP-MM) mismatches in cleaving EGFP allows for the quantification of the editing specificity. While the percentage of EGFP negative cells obtained with the on-target sgRNA quickly reached a plateau at 10 days post infection, the two mismatched sgRNAs generated unspecific EGFP knock-outs which accumulated over time (Supplementary Fig. 1a). The delivery of the recently developed more specific eSpCas9(1.1) variant^18^ guided by the same sgRNAs only partially reversed the time dependent accumulation of off-target cleavages (Supplementary Fig. 1b). Consistently, the analysis of two genomic loci (*ZSCAN* and *VEGFA*) and related off-target sites^19^ indicated that the on/off ratios decreased over time, thus confirming increased off-target cleavages (Supplementary Fig. 1c,d). These results clearly show that the delivery of SpCas9 through a conventional lentiviral system correlates with increased off-target activity and this is particularly evident over time due to prolonged SpCas9 expression.

**Figure 1:**
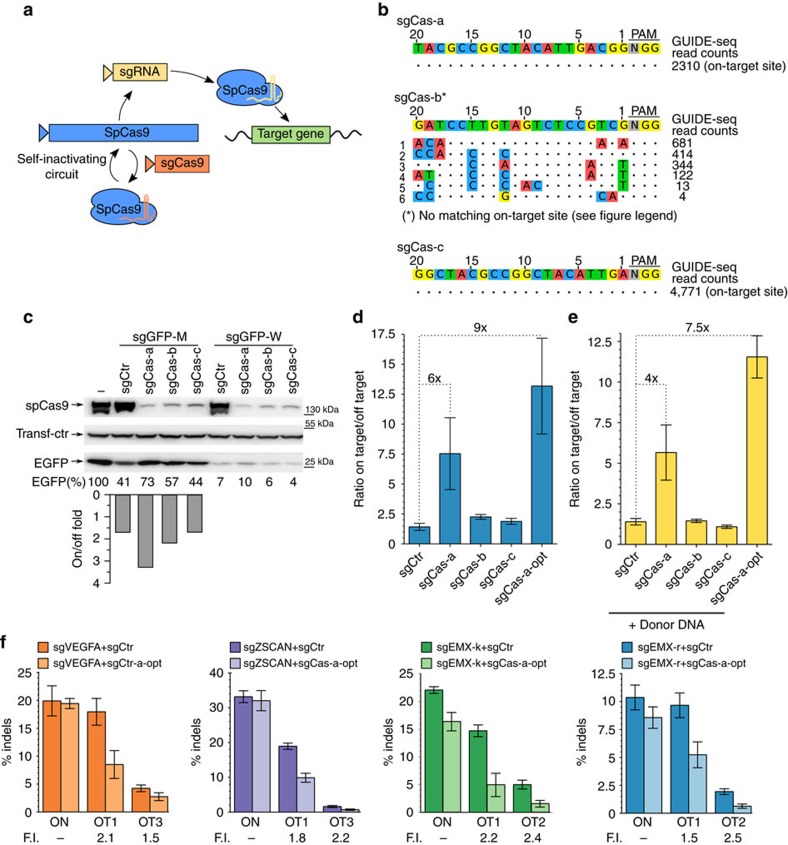
The SLiCES circuit. (**a**) Scheme of the SLiCES circuit. (**b**) GUIDE-seq analysis performed in HEK293T cells stably expressing SpCas9 and transfected with sgCas-a, -b and -c individually. Genomic DNAs obtained from three independent experiments were pooled prior to library preparation. (*) The on-target site was not detected in sgCas-b samples since this guide targets the 3 × FLAG tag fused to SpCas9 which was absent from SpCas9 expressed in 293T-SpCas9 cells. (**c**) Western blot analysis of HEK 293T cells co-transfected with plasmids expressing EGFP, SpCas9 and sgRNAs fully (sgGFP-W) or partially matching (sgGFP-M) the EGFP coding sequence in combination with three sgRNAs targeting the SpCas9 ORF (sgCas-a, -b, -c) or a control sgRNA (sgCtr), as indicated. Lane (−) corresponds to a reference sample containing the non-targeting sgCtr only. Transfection efficiency was normalized using roTag tagged MHC-Iα expression plasmid (Transf-ctr). Lower graph reports the ratio of the percentages of decreased EGFP levels obtained using sgGFP-W (on-target) over the percentages obtained with sgGFP-M (off-target) in the presence of sgCas-a, -b, -c as indicated. (**d**) Target specificity of SpCas9 activity using different SLiCES circuits. On/off ratios were obtained from the percentage of EGFP negative cells after targeting a single chromosomal EGFP gene copy (293-iEGFP cells) with sgGFP-W (on-target) relative to sgGFP-M (off-target) in combination with different SLiCES circuits (sgCas) or a non-targeting (sgCtr) sgRNA, as indicated in the graph. (**e**) Target specificity of SpCas9 activity (on/off ratios) using different self-limiting circuits applied to a gene substitution model (293-iY66S). On/off ratios were obtained from the percentage of EGFP positive cells generated by SpCas9-induced homology-directed repair of the EGFP-Y66S mutation with the sgGFP-M (on-target) relative to the sgGFP-W (off-target) sgRNAs in combination with a DNA donor plasmid (carrying a truncated wild-type EGFP sequence) and the indicated self-targeting sgRNAs. (**f**) Indels formation induced by the SLiCES circuit (sgCas-a-opt) targeting the *VEGFA*, *ZSCAN*, *EMX* loci and their respective validated off-target sites. Fold increase (F.I.) of the on/off ratio with the sgCas-a-opt relative to the sgCtr is reported below the graphs for each off-target. Per cent modification was quantified by TIDE analysis. In **d**–**f** data presented as mean±s.e.m. for *n*≥2 independent experiments.

To generate a transient SpCas9 activity peak in target cells, we developed SLiCES consisting in a SpCas9-sgRNA towards a specific genomic locus combined with a self-limiting sgRNA to switch off the nuclease activity (schematized in Fig. 1a). The self-limiting sgRNAs were designed by *in silico* analysis to target the SpCas9 coding sequence (sgCas-a, -c) and the amino-terminus 3 × FLAG tag fused to SpCas9 (sgCas-b). The SpCas9 coding sequence, after human codon optimization and further modifications (nuclear localization signals and FLAG-tag), is suitable for the design of a variety of non-repetitive sgRNAs with very few predicted off-targets in the human genome (Supplementary Table 1 and Supplementary Data 1). The potential off-target sites generated by sgCas-a, -b and -c were experimentally evaluated in HEK 293T cells stably expressing SpCas9 through GUIDE-seq analysis, a genome-wide unbiased approach^7^,^20^. Both sgCas-a and -c did not generate detectable off-target sites while retaining the ability to efficiently cleave the on-target, while sgCas-b produced at least six off-targets (Fig. 1b and Supplementary Data 2).

The sgCas-a, -b and -c were then evaluated for their SpCas9 self-limiting properties in cells expressing EGFP showing efficient downregulation of SpCas9 when co-expressed with SpCas9 (Fig. 1c, upper panel). Moreover, co-expression of any of the three self-targeting sgRNAs (sgCas-a, -b or -c) together with a sgRNA that fully base pairs with the EGFP target sequence (sgGFP-W) reduced EGFP (4–10% of residual protein) to similar levels as those obtained with sgGFP-W combined with a control sgRNA (sgCtr) (Fig. 1c). These results demonstrate that DNA editing activity is not impaired when SpCas9 is inactivated through the SLiCES circuit. A similar experiment performed using a sgRNA targeting EGFP with a single mismatch within the seed region immediately upstream of the PAM sequence (sgGFP-M) showed non-specific EGFP downregulation, with almost 60% decrease of EGFP intracellular levels. This effect was less pronounced (∼25–40% reduction) in cells where SpCas9 expression was downregulated through the self-limiting Cas9 circuit driven by sgCas-a and -b, while no improvement was observed with sgCas-c (Fig. 1c). The different levels of non-specific EGFP downregulation closely reflected the ability of individual sgRNAs to decrease the intracellular levels of SpCas9: sgCas-a which generated the lowest non-specific EGFP downregulation (73% residual EGFP, Fig. 1c) showed the highest SpCas9 disruption activity (Fig. 1c, upper panel). Similar results were obtained with a reciprocal experiment where cells were transiently transfected with a mutated EGFP target characterized by a single nucleotide substitution (EGFP-Y66S) that fully matched the sgGFP-M sequence (Supplementary Fig. 2).

The improved target specificity of about 2–3 fold (Fig. 1c and Supplementary Fig. 2, lower panel), as defined by the ratio between SpCas9 activity in cells targeted by the perfectly matched sgRNA over the mismatched sgRNA, was also confirmed in 293-iEGFP cells carrying a single chromosomal copy of the *EGFP* gene (sixfold improvement) (Fig. 1d and Supplementary Fig. 3). To test whether the optimization of the sgRNAs may further improve the on-target specificity, the sgRNAs were structurally modified to increase their transcription and interaction with SpCas9 (ref. 21). Optimization of sgCas-a showed a slight decrease of SpCas9 intracellular levels with respect to the non-optimized version, paralleled by improved specificity of the relative SLiCES circuit (about ninefolds) (Fig. 1d and Supplementary Fig. 4). Conversely, the optimization of the sgRNAs towards the target sequence (sgGFP-W/M-opt) did not show improved specificity (Supplementary Fig. 4). Remarkably, while the SLiCES circuit containing sgCas-c did not improve the editing specificity, moderate improvement could be obtained through further downregulation of SpCas9 expression by sgRNA optimization (sgCas-c-opt) (Supplementary Fig. 4). A parallel experiment aimed at validating the on-target specificity of the SpCas9 self-limiting circuit was performed in cells carrying a single chromosomal copy of a non-fluorescent EGFP (Y66S). In these cells, 293-iY66S, SpCas9 activity was measured by the recovery of EGFP fluorescence following the substitution of the mutated gene with a wild-type allele through SpCas9 mediated homology-directed repair in the presence of a co-transfected donor plasmid carrying a non-fluorescent fragment of wild-type EGFP. Compared to the conventional SpCas9 approach (sgCtr), the target specificity for EGFP homology-directed repair was improved by using the SLiCES circuit (sgCas-a) by fourfold (Fig. 1e and Supplementary Fig. 5). Further improvement (7.5-fold) was obtained with the optimized version of sgCas-a (sgCas-a-opt) (Fig. 1e and Supplementary Fig. 5), as previously observed in knock-out experiments.

To demonstrate that the SLiCES methodology is readily transferrable to other RNA-guided nucleases, SLiCES was adapted to Cas9 from *Streptococcus thermophilus* (St1Cas9) by using specific sgRNAs (sgCas-St1-1, -2 and -3) to induce St1Cas9 downregulation (Supplementary Fig. 6). The SLiCES circuits carrying sgCas-St1-1 and -3 resulted in an improved St1Cas9 cleavage specificity by, respectively, 3.4- and 1.9-folds (Supplementary Fig. 6). These results indicate the versatility of the SLiCES circuit towards nucleases other than SpCas9 including new emerging variants^18^,^19^,^22^,^23^,^24^.

Next, the target specificity of the conventional SpCas9 and the SLiCES circuit (sgCas-a-opt) towards endogenous sequences was comparatively analysed. Four genomic sites (*VEGFA*, *ZSCAN* and two targets in the *EMX* locus) and two previously validated off-target sites^19^ for each sgRNA were analysed by tracking indels by decomposition (TIDE) (ref. 25) revealing that the SLiCES approach improved cleavage specificity by ∼1.5–2.5-folds (Fig. 1f).

### Lentiviral delivery of the SLiCES circuit

**Figure 2:**
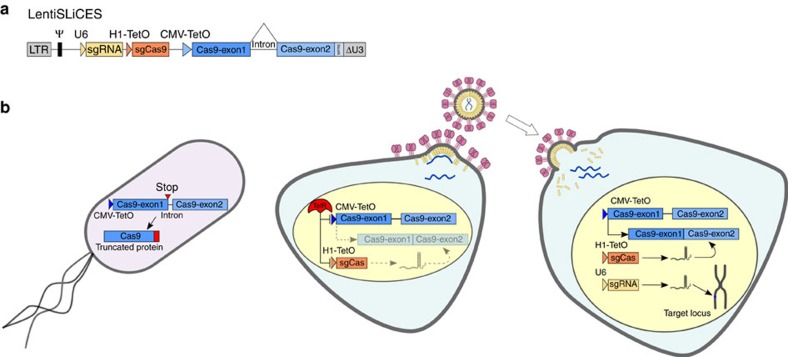
The lentiSLiCES system. (**a**) Graphical representation of lentiSLiCES viral vector. (**b**) Steps required for the production of the lentiSLiCES viral vectors. SpCas9 expression is prevented in bacterial cells to allow plasmid amplification through the introduction of a mammalian intron within the SpCas9 open reading frame. Production of lentiSLiCES viral particles is obtained in cells stably expressing the Tetracycline Repressor (TetR) to prevent SpCas9 and sgCas self-limiting sgRNA expression driven by Tet repressible promoters. In target cells the absence of the TetR allows the expression of the lentiSLiCES circuit leading to target genome editing and simultaneous SpCas9 downregulation.

**Figure 3:**
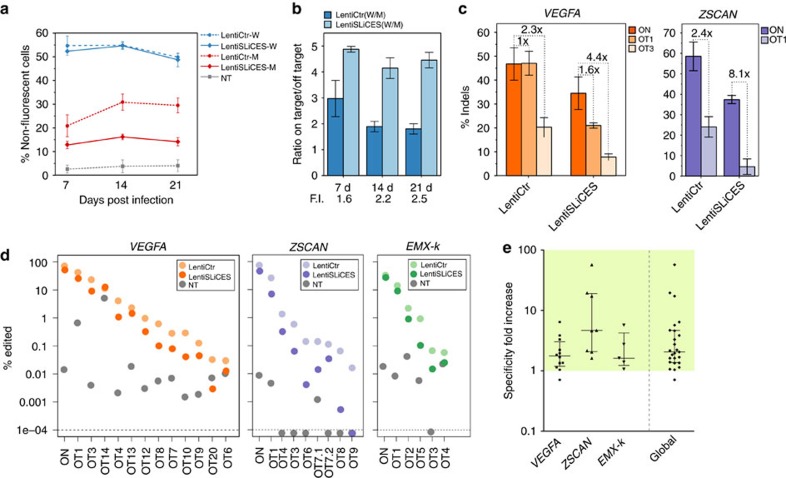
Genome editing with lentiSLiCES vectors. (**a**) EGFP knock-down by lentiSLiCES vectors. Time-course curves of the percentages of EGFP negative 293-multiEGFP cells, following transduction with lentiviral vectors carrying self-targeting (lentiSLiCES) or non-self-targeting (lentiCtr) sgRNAs in combination with either sgGFP-W (on-target) or sgGFP-M (off-target) sgRNAs, as indicated in the graph. Data presented as mean±s.e.m. for *n*=2 independent experiments. (**b**) Target specificity of SpCas9 delivered through the lentiSLiCES. On/off ratios were calculated from the percentages of EGFP negative cells reported in **a**. The F.I. of specificity calculated from the on/off ratios at each time point is reported below the graphs. Data presented as mean±s.e.m. for *n*=2 independent experiments. (**c**) Indels formation induced by lentiSLiCES vectors at the *ZSCAN* and *VEGFA* loci and at their previously validated off-target sites. Per cent modification was quantified by TIDE analysis on genomic DNA collected 20 days post transduction and selection with blasticidin. Values indicate the on/off ratios calculated from indels obtained with each off target. Data presented as mean±s.e.m. for *n*=2 independent experiments. (**d**) Target specificity of lentiSLiCES in primary human fibroblasts. Targeted deep-sequencing analysis of indel formation at the on-target site and at previously validated off-targets of the *VEGFA, ZSCAN* and *EMX-k* genomic loci induced by SpCas9 delivered through lentiSLiCES or lentiCtr. Genomic DNAs were extracted at 20 days post transduction and selection with blasticidin. NT indicates the background level of indels measured in non-edited cells. Genomic DNAs obtained from two independent experiments were pooled prior to amplicon preparation. (**e**) Specificity F.I. of lentiSLiCES obtained by dividing the on/off ratios of lentiSLiCES and lentiCtr (see Supplementary Data 3) calculated for each locus from **d**. The median and interquartile range are shown. More than 100,000 sequences were analysed per on-target sample and an average of 143,000 sequences were analysed per off-target sample (Supplementary Data 3). The observed values equal to 0 were approximated to 1e-05. The green-shaded area indicates a ratio above one, which corresponds to an increase in the on/off ratio.

The self-limiting SpCas9-sgRNA circuit with the best performing self-limiting sgRNA (sgCas-a-opt) was then transferred to a lentiviral system (Fig. 2) to generate lentiSLiCES. To avoid the leaky expression of SpCas9, and the consequent degradation of DNA during plasmid preparation in bacteria, an intron was introduced into the SpCas9 open reading frame to form an expression cassette divided in two exons (exon 1 and 2, schematized in Fig. 2). As splicing does not occur in bacteria, the transcripts produced are translated in these cells as a catalytically inactive SpCas9 fragment. Next, to circumvent the self-cleavage activity during lentiviral vector production, Tetracycline inducible (TetO) promoters were introduced to regulate both SpCas9 and the self-targeting sgRNAs expression. The TetO promoter is negatively regulated by a specific repressor, Tet repressor (TetR), which is expressed in producing cells, and in the absence of doxycycline, inhibits transcription through its binding to tetracycline operator sequences located within the promoter region (schematized in Fig. 2b). The drop in SpCas9 intracellular levels in producing cells observed with the activation of the self-limiting circuit with doxycycline demonstrates the strict requirement of the repressible promoters at viral production steps in order to obtain un-altered lentiSLiCES particles (Supplementary Fig. 7a). Furthermore, the relevance of the TetR-mediated repressible conditions during vector production was clear from the analysis of the SpCas9 transgene sequence which was highly modified in lentiSLiCES particles produced in the absence of TetR-mediated repression; these particles showed poor editing capacity (Supplementary Fig. 7b). To evaluate the on-/off-target activity of the lentiSLiCES, the percentage of EGFP negative 293-multiEGFP cells was followed at different time points after transduction with self-limiting lentiviral vectors either carrying the specific sgRNA sgGFP-W (lentiSLiCES-W) or the mismatched sgGFP-M (lentiSLiCES-M) and compared with the effect obtained with non-self-limiting lentiviral vectors carrying the same sgRNAs (lentiCtr-W or -M). Both lentiCtr-W and lentiSLICES-W showed similarly stable on-target activity at all the time points within a 3 weeks period (Fig. 3a). Conversely, the percentage of EGFP cells unspecifically targeted by the sgGFP-M increased over time with the lentiCtr delivery system; this effect was not observed with the same sgRNA delivered through lentiSLiCES throughout the 3 weeks period (Fig. 3a). Therefore, lentiSLiCES generated no off-target accumulation over time (compare day 7 and day 21, Fig. 3b). Consistently, at the end-point we observed the largest difference between the ratios of the EGFP negative cells obtained with the sgGFP-W over the sgGFP-M delivered either through the lentiSLICES (on/off ratio ∼5) or the lentiCtr systems (on/off ratio ∼2) (Fig. 3b). In agreement with these results the target specificity of the lentiSLiCES towards endogenous sequences (*ZSCAN* and *VEGFA* loci) showed significant improvement as compared to the non-self-limiting lentiCtr (∼2–4-fold) (Fig. 3c).

The editing properties of lentiSLiCES were finally tested in non-transformed cell lines. Primary fibroblasts were transduced with lentiSLiCES directed towards the *VEGFA*, *ZSCAN* and *EMX-k* loci and tested for on-/off-target cleavages through targeted deep-sequencing. The analysis revealed that out of 25 tested off-target sites only at one site (OT14 in the *VEGFA* locus) lentiSLiCES generated slightly more unspecific cleavages than the conventional lentiCtr approach (Fig. 3d and Supplementary Data 3). Similarly, in human neural progenitor cells the editing of the *VEGFA* locus was more accurately edited by using the lentiSLiCES approach (Supplementary Fig. 8). Overall, the downregulation of SpCas9 expression through the self-limiting lentiSLiCES circuit decreased the off-target events in 24 out of 25 analysed off-target sites associated with three genomic loci (*VEGFA*, *ZSCAN* and *EMX-k*) with a median specificity fold improvement equal to 2.06 (25th and 75th percentile equal to 1.37 and 4.65, respectively) (Fig. 3e and Supplementary Data 3).

### Hit and Go Cas9 delivered through lentiSLiCES

**Figure 4:**
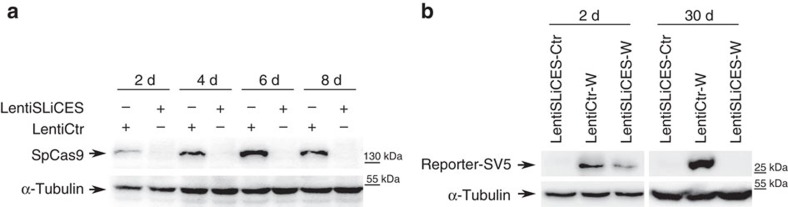
SpCas9 expression and activity in lentiSLiCES transduced cells. (**a**) Expression levels of SpCas9 at the indicated time points after transduction with lentiSLiCES or with lentiCtr. SpCas9 was detected using an anti-FLAG antibody. Western blot is representative of *n*=2 independent experiments. (**b**) SpCas9 activity monitored by SV5-EGFP protein levels produced by the NHEJ-reporter plasmid transfected in 293-multiEGFP cells before or 28 days after transduction and detected at 2 days or 30 days post transduction, as indicated, with lentiSLICES targeting (lentiSLiCES-W) or non-targeting EGFP (lentiSLiCES-Ctr). The activity of the non-self-limiting lentiCtr-W vector targeting EGFP was monitored at the same time points for comparison. Western blot is representative of *n*=2 independent experiments.

To directly prove the transient nature of SpCas9 expression induced by lentiSLiCES, we measured the SpCas9 intracellular levels obtained following transduction. SpCas9 delivered through a non-self-limiting lentiviral system (lentiCtr) was detected as early as 2 days post transduction and further increased at subsequent time points (Fig. 4a). Conversely, in cells transduced with lentiSLiCES low levels of Cas9 were detected only at 2 days post transduction and decreased below the detection limit at subsequent time points (Fig. 4a). Finally, to functionally assess the level of SpCas9 activity delivered through the lentiSLiCES, a non-homologous end joining (NHEJ) reporter plasmid (NHEJ-Rep.W) expressing the simian virus-5 tag fused with EGFP (SV5-EGFP) upon targeted nuclease activity (schematized in Supplementary Fig. 6a) was employed. The NHEJ-Rep.W revealed that SpCas9 delivered through the lentiCtr was active at all time points following transduction, while the activity of SpCas9 carried by the lentiSLiCES was detected 2 days after transduction, but could not be observed at later time points (30 days) (Fig. 4b). These data demonstrate that SpCas9 nuclease activity is abrogated following its transduction and genome modification in target cells.

## Discussion

Genome editing through CRISPR-Cas9 technology is a revolutionary approach opening new perspectives towards the development of novel therapeutic protocols^12^. This technology has been used with success both *in vitro* as well as in animal models^26^. Nevertheless, the incomplete control on CRISPR-Cas9 specificity^4^,^5^,^6^,^7^,^8^,^9^,^10^,^11^ raises significant concerns on its clinical use^27^. In line with these concerns our data clearly show that long-term nuclease expression delivered through lentiviral systems, used for efficient *in vivo* delivery, results in the accumulation of unwanted cleavages. This detrimental effect could not be overcome even with the recently developed, more specific SpCas9 variant, eSpCas9(1.1) (ref. 18). Transient SpCas9 expression for genome editing applications can be obtained by RGENs direct delivery^15^,^16^,^17^, however this methodology is severely limited by the low efficiency in the percentages of targeted cells. Conversely, our self-limiting circuit strategy, lentiSLiCES, exploits the efficiency of viral-based delivery and simultaneously limits the amount of SpCas9 expressed post transduction. By limiting in time and abundance the intracellular levels of Cas9, SLiCES avoids the accumulation of off-target cleavages that instead are observed with the use of conventional Cas9 delivery approaches^14^,^15^,^28^. Beyond, limited off-target activity, the main advantage of the SLiCES approach is a nuclease-free cellular environment following genome modification which greatly improves the safety margins for this technology. The transient nature of SpCas9 may also be non trivial for applications such as genetic screening where unspecific binding of SpCas9 to non-cleaved sites may alter the final outcome of the screening^28^,^29^. The pre-existing immune response against SpCas9 recently proved in mice suggests that *in vivo* approaches will be severely limited by the immune response against this protein^11^, thus the transient nature of the SLiCES system may have a significant impact for the clinical use.

To further improve the SLiCES strategy, Integrase Defective Lentiviral Vectors (IDLV)^30^ could be used to maintain the viral-based efficiency in cellular delivery, while enhancing the transient peak-like nature of Cas9 expression. Other episomal viral systems exploitable to preserve the ‘hit and go' nature of the SLiCES approach include the AAV vectors. These are vectors reported to efficiently deliver small Cas9 variants (such as SaCas9)^31^, that can be potentially adapted to an ‘all-in-one' AAV-SLiCES vector. Alternatively, AAV-mediated SLiCES delivery for large size nucleases, such as SpCas9 used in this study or AsCpf1, can be obtained through a co-infection strategy^32^, where the nucleases are separated from the sgRNAs transfer vector. The use of episomal viral delivery systems (AAV or IDLV) may improve the SLiCES approach by circumventing potential cleavages of the integrated SpCas9 sequence by residual non-edited nuclease. In fact, SpCas9 sequence harboured in episomes could in principle prevent possible genomic damages which may lead to chromosomal rearrangement^33^. A variety of Cas9 applications, such as the regulation of gene expression obtained by the combination with transcriptional activation domains^34^,^35^,^36^ might be significantly improved through their adaptation to lentiSLiCES. In fact, these approaches as well as the refined modulation of gene expression obtained with a genetic kill-switch circuit^37^,^38^ could be potentiated by a tunable self-limiting approach to restrict in time Cas9-mediated induction of the targeted cellular promoters. Finally, SLiCES may significantly improve some recently developed Cas9 genome engineering procedures that are susceptible to continuous nuclease activity. For instance, current techniques to efficiently substitute genomic sequences use Cas9 to increase the rate of homology-directed repair^1^; nevertheless, these techniques are often limited by the continuous re-cleavage of the newly substituted genomic sequence by Cas9 (ref. 39), which could be easily overcome by nuclease inactivation.

Similar approaches aimed at controlling Cas9 activity have been recently developed by exploiting various inducible systems^40^. Nevertheless, the approaches reported so far suffer of a number of limitations spanning from decreasing editing activity generated by nuclease splitting^41^ or chemical modification^14^ to background activity^42^ or extended time of required induction^43^.

Overall, the ‘hit and go' nature of SLiCES and its adaptability to new emerging Cas9 techniques, combined with the implementation of its viral delivery, allows for more controllable genome editing procedures with limited unwanted off-target activity.

## Methods

### Plasmids and oligonucleotides

The 3 × FLAG-tagged SpCas9 was expressed from the pX-Cas9 plasmid, which was obtained by removal of an NdeI fragment including the sgRNA expression cassette from pX330 (a gift from Feng Zhang, Addgene # 42230) (ref. 1). The sgRNAs were transcribed from a U6 promoter driven cassette, derived from pX330 and cloned into pUC19. sgRNA oligos were cloned using a double BbsI site inserted before the sgRNA constant portion^1^. Plasmids expressing FLAG-tagged St1Cas9 (pJDS246-CMV-St1-Cas9) and St1Cas9 *gRNA* (pMLM3636-U6-+103gRNA_St1Cas9) were a gift of Claudio Mussolino^44^. St1Cas9 sgRNAs oligos were cloned into pMLM3636-U6-+103gRNA_St1Cas9 using BsmBI and transcribed from a U6 promoter. The list of sgRNAs and target sites employed in this study is available in Supplementary Data 4.

pcDNA5-FRT-TO-EGFP plasmid was obtained by subcloning EGFP from pEGFP-N1 in a previously published vector^45^ derived from pcDNA5-FRT-TO (Invitrogen). pcDNA5-FRT-TO-EGFP-Y66S was obtained by site directed mutagenesis of pcDNA5-FRT-TO-EGFP. A sgRNA resistant, non-fluorescent truncated EGFP fragment (1-T203K-stop), obtained by site directed mutagenesis of the pcDNA5-FRT-TO-EGFP plasmid, was amplified by PCR and inserted in place of EGFP in the pcDNA5-FRT-TO-EGFP plasmid, yielding the donor pcDNA5-FRT-TO-rEGFP(1-T203K-stop) plasmid.

The SV5-EGFP-based NHEJ reporters employed in this study (Rep. SV5, NHEJ-REP.W and NHEJ-Rep.M) were generated by cloning into the NheI and BspEI sites dsDNA oligos corresponding to the complete target sequence (including PAM) recognized by a sgRNA of interest. The target is inserted between the SV5 tag and EGFP coding sequences, with the EGFP sequence positioned out of frame with respect to the starting ATG codon of the SV5 tag open reading frame (ORF). A stop codon is inserted in the SV5 frame, immediately after the target sequence. The pcDNA3 MHC-I-roTag plasmid is described in ref. 46. Information on plasmids DNA sequences produced for this manuscript are found in Supplementary Figs 9 and 10.

### Cell culture and transfections

HEK 293T/17 cells were obtained from ATCC. 293TR cells, constitutively expressing the TetR, were generated by lentiviral transduction of parental HEK 293T/17 cells using the pLenti-CMV-TetR-Blast vector (a gift from Eric Campeau, Addgene # 17492) (ref. 47) and were pool-selected with 5 μg ml^−1^ of blasticidin (Life Technologies). Similarly, 293T-SpCas9 cells, stably expressing SpCas9, were obtained by lentiviral transduction with the lentiCas9-Blast vector (a gift from Feng Zhang, Addgene # 52962) (ref. 48 at MOI of one and were pool-selected using 5 μg ml^−1^ of blasticidin. 293-multiEGFP cells were generated by stable transfection of pEGFP-IRES-Puromicin and selected with 1 μg ml^−1^ of puromicin. 293-iEGFP and 293-iY66S cells (Flp-In T-REx system; Life Technologies) were generated by Flp-mediated recombination using the pcDNA5-FRT-TO-EGFP or the pcDNA5-FRT-TO-EGFP-Y66S as donor plasmids, respectively, in cells carrying a single genomic FRT site and stably expressing the tetracycline repressor (293T-Rex Flp-In, cultured in selective medium containing 15 μg ml^−1^ blasticidin and 100 μg ml^−1^ zeocin-Life Technologies). 293-iEGFP and 293-iY66S were cultured in selective medium containing 15 μg ml^−1^ blasticidin and 100 μg ml^−1^ hygromycin (Life Technologies). 293-iEGFP and 293-iY66S selected clones were checked for integration specificity by loss of zeocin resistance. Untransformed human primary fibroblasts (GM03815) were obtained from Coriell Cell Repositories. All the above cell lines were cultured in DMEM supplemented with 10% FBS, 2 mM L-Gln, 10 U ml^−1^ penicillin and 10 μg ml^−1^ streptomycin and the appropriate antibiotics indicated above. The neural progenitor cell line AF22 cells were kindly donated by Austin Smith (University of Cambridge, UK). The cells are maintained as previously described^49^. Briefly, cells were cultured in DMEM/F:12 medium (Life Technologies) supplemented with 1% N2 Supplement (Life Technologies, Cod. 17502048), 0.1% B27 Supplement (Life Technologies), 10 ng ml^−1^ EGF (Peprotech) and bFGF (Peprotech).

HEK 293T, 293-iEGFP or 293-iY66S cells were transfected in 12 or 24 multi wells with 250–500 ng of pX-Cas9 and 250–500 ng of the desired pUC19-sgRNA plasmid using TransIT-LT1 (Mirus Bio), according to manufacturer's instructions. Cells were collected 2–4 days after transfection or as indicated.

In 293-iEGFP and 293-iY66S cells the expression of EGFP was induced by treatment with 100 ng ml^−1^ doxycycline (Cayman Chemical) for 20 h before fluorescence measurement.

All cell lines were verified mycoplasma-free (PlasmoTest, Invivogen).

### lentiSLiCES vectors

lentiSLiCES was prepared from lentiCRISPRv1 transfer vector^28^ by substituting the EFS-SpCas9-2A-Puro cassette with a SpCas9(intron)-IRES-Blasticidin fragment together with a CMV-TetO promoter. The intron introduced in SpCas9 (see Supplementary Fig. 11) derives from the mouse immunoglobulin heavy chain precursor V-region intron (GenBank ID: M12880.1), previously used with different flanking exons^45^,^46^,^50^. The EMCV-IRES regulating the translation of a blasticidin resistance gene was cloned downstream of SpCas9 to allow the antibiotic selection of transduced cells, even after the generation of frameshift mutations following Cas9 self-cleavage of the integrated vector.

The sgCtr-opt or the sgCas9-a-opt were assembled with an H1-TetO promoter within the pUC19 plasmid, PCR amplified and then cloned into a unique EcoRI site in lentiCRISPRv1 and selected for the desired orientation. The sgRNAs targeting the chosen locus were cloned into the lentiCRISPRv1 sgRNA cassette using the two BsmBI sites, following standard procedures^28^.

Information on DNA sequences of lentiSLiCES can be found in Supplementary Information.

### Lentiviral vector production

Lentiviral particles were produced by seeding 4 × 10^6^ HEK 293T or 293TR cells into a 10 cm dish, for lentiCRISPR or lentiSLiCES production, respectively. The day after the plates were transfected with 10 μg of each transfer vector together with 6.5 μg pCMV-deltaR8.91 packaging vector and 3.5 μg pMD2.G using the polyethylenimine (PEI) method^51^. After an overnight incubation, the medium was replaced with fresh complete DMEM and 48 h later the supernatant containing the viral particles was collected, spun down at 500*g* for 5 min and filtered through a 0.45 μm PES filter.

After collection, lentiSLiCES viral vectors were concentrated using polyethylene glycol (PEG) 6,000 (Sigma). Briefly, a 40% of PEG 6,000 solution in water was mixed in a 1:3 ratio with the vector-containing supernatant and incubated for 3 h to overnight at 4 °C. Subsequently, the mix was spun down for 45 min at 2,000*g* in a refrigerated centrifuge. The pellets were then resuspended in a suitable volume of DMEM complete medium. lentiCRISPR vectors were used unconcentrated. The titre of the lentiviral vectors (reverse transcriptase units, RTU) was measured using the product enhanced reverse transcriptase (PERT) assay^52^.

### Infections and EGFP fluorescence detection

One day before transduction 10^5^ HEK 293T, 293-iEGFP or 293-multiEGFP cells or 40,000 fibroblasts were seeded in a 24-well plate. For lentiSLiCES vectors, cells were transduced by centrifuging 2 RTU per well for 2 h at 1,600*g* at 16 °C, and then leaving the vectors incubating with the cultures for an overnight. Starting from 24 h post transduction onwards the cultures were selected with 5 μg ml^−1^ of blasticidin, where needed. For lentiCRISPR vectors, 0.5 RTU per well were used following the same transduction protocol and cells were selected with 0.5 μg ml^−1^ of puromycin.

For infection experiments using neural progenitor cells, 40,000 cells per cm^2^ were plated on laminin coated 24-wells the day before infection. Infection was performed by centrifuging 2 RTU per well of each vector on the cells at 500*g* for 30 min RT. Medium was changed completely 16 h post infection to remove the viral particles and 48 h later the cells were treated using 20 μg ml^−1^ of blasticidin for positive selection of the transduced cells. Cells were maintained under selection for 30 days and detached from tissue culture flasks with Accutase solution (Sigma) when reached the confluence.

When targeting genomic EGFP sequences, cells were collected and analysed using a FACSCanto flow cytometer (BD Biosciences) to quantify the percentage of EGFP loss or induction (gene substitution experiments).

### Western blots

Cells were lysed in NEHN buffer (20 mM HEPES pH 7.5, 300 mM NaCl, 0.5% NP40, NaCl, 1 mM EDTA, 20% glycerol supplemented with 1% of protease inhibitor cocktail (Pierce). Cell extracts were separated by SDS-PAGE using the PageRuler Plus Protein Standards as the standard molecular mass markers (Thermo Fisher Scientific). After electrophoresis, samples were transferred to 0.22 μm PVDF membranes (GE Healthcare). The membranes were incubated with mouse anti-FLAG M2 (F1804, Sigma, dilution 1:10^3^) for detecting SpCas9 and St1Cas9, mouse anti-α-tubulin DM1A (T9026, Sigma, dilution 1:10^3^), rabbit anti-GFP (sc8334, Santa Cruz Biotechnology, dilution 1:10^3^), mouse anti-roTag mAb^46^ (hybridoma culture supernatant) and with the appropriate HRP conjugated goat anti-mouse IgG+M (H+L) (5210-0188 01-18-09, KPL, dilution 1:10^4^) or goat anti-rabbit IgG (sc-2030, Santa Cruz Biotechnology, 1:5,000) secondary antibodies for ECL detection. Images were acquired and bands were quantified using the UVItec Alliance detection system. Uncropped blot images are reported in Supplementary Fig. 12.

### Detection of Cas9-induced genomic mutations

Genomic DNA was isolated at 72 h post transfection or as indicated for transduction experiments, using the DNeasy Blood & Tissue kit (Qiagen). PCR reactions to amplify genomic loci were performed using the Phusion High-Fidelity DNA polymerase (Thermo Fisher). Samples were amplified using the oligos listed in Supplementary Data 5. Purified PCR products were analysed either by sequencing and applying the TIDE tool^25^ or by T7 Endonuclease 1 (T7E1) assay (New England BioLabs). In the latter case PCR amplicons were denatured and re-hybridized before digestion with T7E1 for 30 min at 37 °C. Digested material was separated using standard agarose gel and quantified using the ImageJ software. Indel formation was calculated according to the following equation: % gene modification=100 × (1−(1–fraction cleaved)^1/2^).

To perform a sequence analysis of the SpCas9 transgene generated at lentiSLiCES production steps, viral vectors were produced in presence and absence of doxycycline. Since, contamination of the original plasmids used for vector production was repeatedly detected in vector supernatants even following DNase treatments, the SpCas9 transgene was analysed at early time points (16 h, at completion of reverse transcription) in 293TR transduced cells. 293TR cell by expressing TetR prevent SLiCES activation and subsequent auto-cleavage of the SpCas9 transgene. Lentiviral cDNA was specifically amplified using a forward oligo in the deltaU3 region and a reverse oligo in the SpCas9 gene (Supplementary Data 5).

### GUIDE-seq experiments and data analysis

GUIDE-seq was performed as previously described^20^ with few modifications. Briefly, HEK 293T cells stably expressing SpCas9 were transfected with 250 ng of sgRNA-encoding plasmid and 10 pmol of annealed GUIDE-seq oligonucleotides (dsODNs) using Lipofectamine 3,000 transfection reagent (Invitrogen). Four days post transfection genomic DNA was extracted using the DNeasy Blood and Tissue kit (Qiagen) following the manufacturer's instructions and sheared to an average length of 500 bp with the Bioruptor Pico sonication device (Diagenode). Library preparations were performed with the original adapters and primers according to previous work^7^. Libraries were quantified with the Qubit dsDNA High Sensitivity Assay kit (Invitrogen) and sequenced with the MiSeq sequencing system (Illumina) using an Illumina Miseq Reagent kit V2–300 cycles (2 × 150 bp paired-end).

Raw sequencing data (FASTQ files) were analysed using the GUIDE-seq computational pipeline^7^. After demultiplexing, putative PCR duplicates were consolidated into single reads. Consolidated reads were mapped to the human reference genome GrCh37 using BWA-MEM^53^; reads with mapping quality lower than 50 were filtered out. Upon the identification of the genomic regions integrating double-stranded oligodeoxynucleotide (dsODNs) in aligned data, RGN sites were retained if at most eight mismatches against the target were present and if absent in the background controls. Visualization of aligned off-target sites is available as a colour-coded sequence grid.

### Target deep-sequencing

Selected off-target sites for the *VEGFA*, *EMX-k* site and *ZSCAN* genomic loci, together with their relative on-target, were amplified using the Phusion high-fidelity polymerase (Thermo Scientific) or the EuroTaq polymerase (Euroclone) from genomic DNA extracted from primary fibroblasts at 20 days after transduction with lentiSLiCES or lentiCtr. Off-target amplicons were pooled in near-equimolar concentrations before purification and indexing. Libraries were indexed by PCR using Nextera indexes (Illumina), quantified with the Qubit dsDNA High Sensitivity Assay kit (Invitrogen), pooled according to the number of targets and sequenced on an Illumina Miseq system using an Illumina Miseq Reagent kit V3–150 cycles (150 bp single read). The complete primer list used to generate the amplicons is reported in Supplementary Data 5.

A reference genome was built using Picard ( http://broadinstitute.github.io/picard) and samtools^54^ from DNA sequences of the considered on-/off-target regions. Raw sequencing data (FASTQ files) were mapped against the created reference genome using BWA-MEM (ref. 53) with standard parameters and resulting alignment files were sorted using samtools. Only reads with mapping quality above or equal to 30 were retained. Presence of indels in each read for each considered region was determined by searching indels of size 1 bp directly adjacent to the predicted cleavage site or indels of size ≥2 bp overlapping flanking regions of size 5 bp around the predicted cleavage site.

### Code availability

Indels identification in targeted deep-sequencing data analysis was performed implementing a script in the R language that is available upon request.

### Data availability

Guide-seq and deep-sequencing data has been deposited at BioProject (https://www.ncbi.nlm.nih.gov/bioproject/) under the accession code PRJNA381704. All other relevant data are available from the authors upon request.

## Additional information

**How to cite this article:** Petris, G. *et al*. Hit and go CAS9 delivered through a lentiviral based self-limiting circuit. *Nat. Commun.* **8,** 15334 doi: 10.1038/ncomms15334 (2017).

**Publisher's note:** Springer Nature remains neutral with regard to jurisdictional claims in published maps and institutional affiliations.

## Supplementary Material

Supplementary Information Supplementary figures and supplementary table.

Supplementary Data 1 Mismatched sites in the reference human genome for the self-targeting sgCas-a determined by Cas-OFFinder software (http://www.rgenome.net/cas-offinder/new)

Supplementary Data 2 GUIDE-seq results for the self-targeting sgRNAs and sequencing statistics for the GUIDE-seq experiments.

Supplementary Data 3 Targeted deep-sequencing results (percentage of indels) on selected off-target sites of the EMX1, VEGFA and ZSCAN2 loci in primary fibroblasts and sequencing statistics.

Supplementary Data 4 Sequences of oligonucleotides used to construct sgRNA expression plasmids and sequences of relative target sites.

Supplementary Data 5 Sequences of the oligos used to amplify EGFP, lentiSpCas9, the genomic loci (VEGFA, ZSCAN, EMX) and relative off-target sites.
