# Supplementary material for: Hit and go CAS9 delivered through a lentiviral based self-limiting circuit
Source: Nat Commun. 2017 May 22;8:15334. doi: 10.1038/ncomms15334 (PMC5458126; doi:10.1038/ncomms15334)
Supplement: Supplementary Information — Supplementary figures and supplementary table. [file ncomms15334-s1.pdf]

SUPPLEMENTARY FIGURES

Supplementary Figure 1

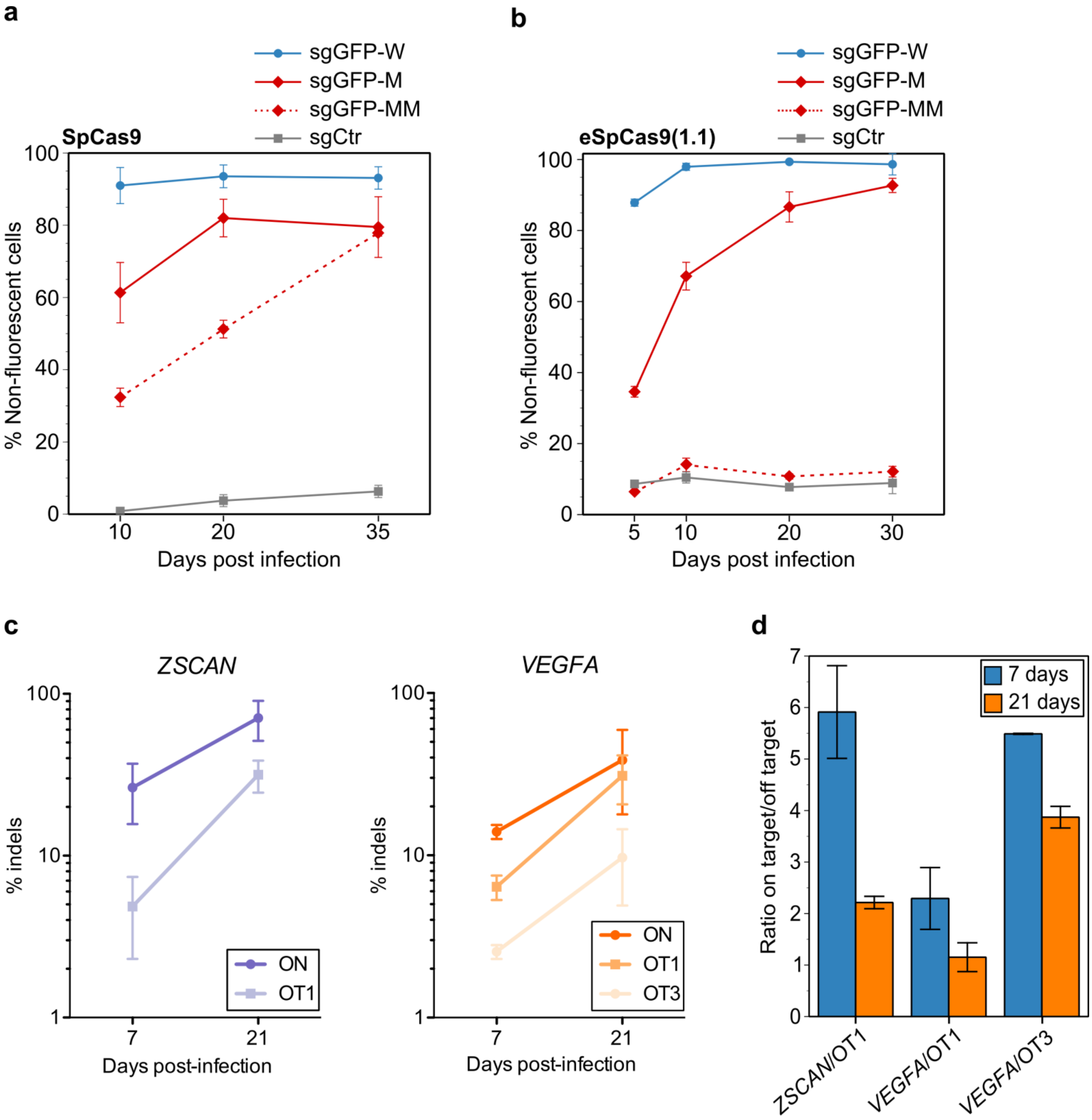

Supplementary Figure 1. Long term expression of Cas9 delivered through a lentiviral vector correlates with the accumulation of off-target cleavages. (**a**) Time course curves of the percentages of 293-iEGFP non-fluorescent cells obtained by the transduction with a lentiviral vector (lentiCRISPR) expressing SpCas9 together with either a perfectly matching sgRNA (sgGFP-W) or two different sgRNAs containing one or two mismatches with the target sequence (sgGFP-M and –MM, respectively). A vector expressing an irrelevant sgRNA was used as control (sgCtr). (**b**) As in (**a**) using a lentiviral vector expressing a SpCas9 variant with increased fidelity (eSpCas9(1.1)). (**c**) Percentage of indels identified after long-term transduction of HEK293T cells with a lentiviral vector (lentiCRISPR) expressing SpCas9 together with sgRNAs targeting the *ZSCAN* and *VEGFA* genomic loci. The indels were analyzed by TIDE at 7 and 21 days post-transduction. (**d**) Data from panel (**c**) expressed as on-target/off-target indels frequency ratio. For all the experiments, cells were selected with puromycin in order to eliminate the non-transduced cells. Data in panels (**a**-**d**) presented as mean $\pm$ s.e.m. for n=2 independent experiments.

Supplementary Figure 2

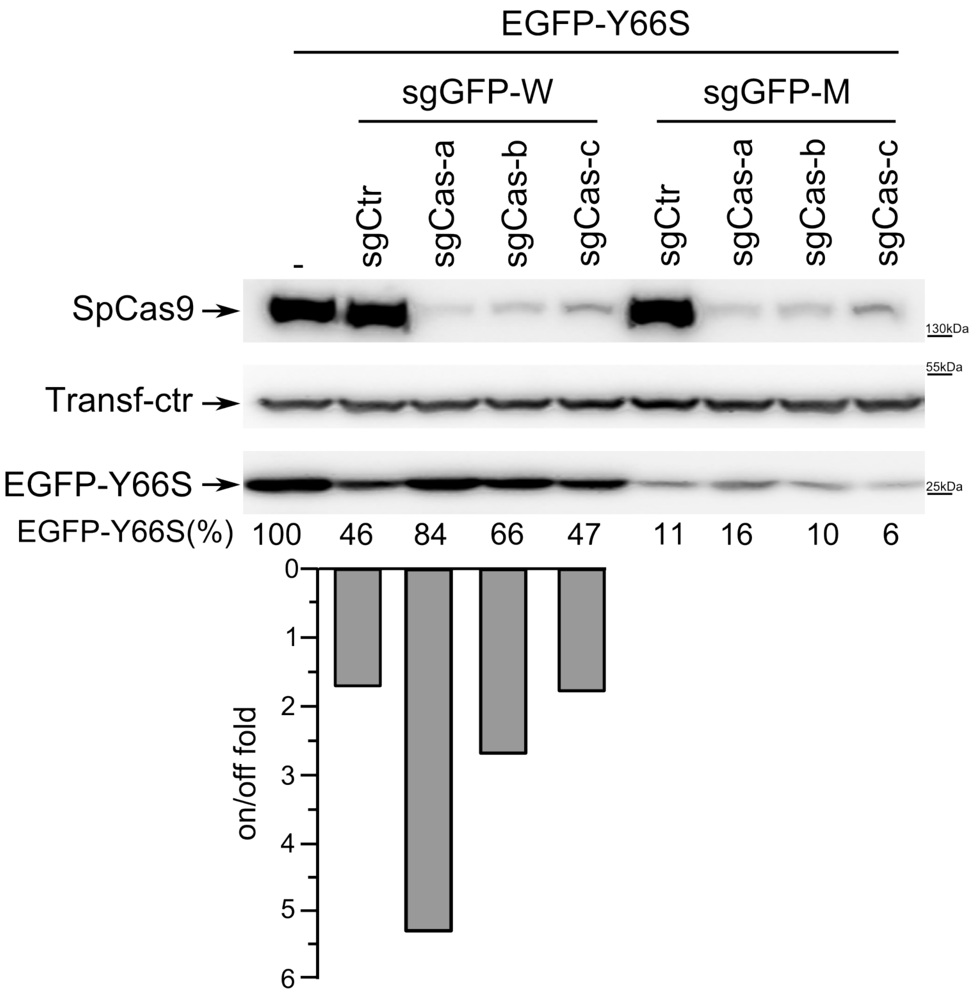

Supplementary Figure 2. Regulation of SpCas9 and EGFP-Y66S expression by the SLiCES circuit. Western blot of cells co-transfected with plasmids expressing EGFP-Y66S, SpCas9, sgRNAs perfectly matching (sgGFP-M) or containing one mismatch (sgGFP-W) with the EGFP-Y66S target sequence together with sgRNAs specific for the SpCas9 ORF (sgCas-a, -b, -c) or a control sgRNA (sgCtr), as indicated. Lane (-) corresponds to a reference sample containing the non-targeting sgCtr only. Transfection efficiency was normalized using roTag

tagged MHC-Iα expression plasmid (Transf-ctr). SpCas9 was detected using an anti-FLAG antibody. Lower graph reports the ratio of the percentages of decreased EGFP-Y66S levels obtained using sgGFP-M (on-target) over the percentages obtained with sgGFP-W (off-target) in the presence of sgCas-a, -b, -c, as indicated.

Supplementary Figure 3

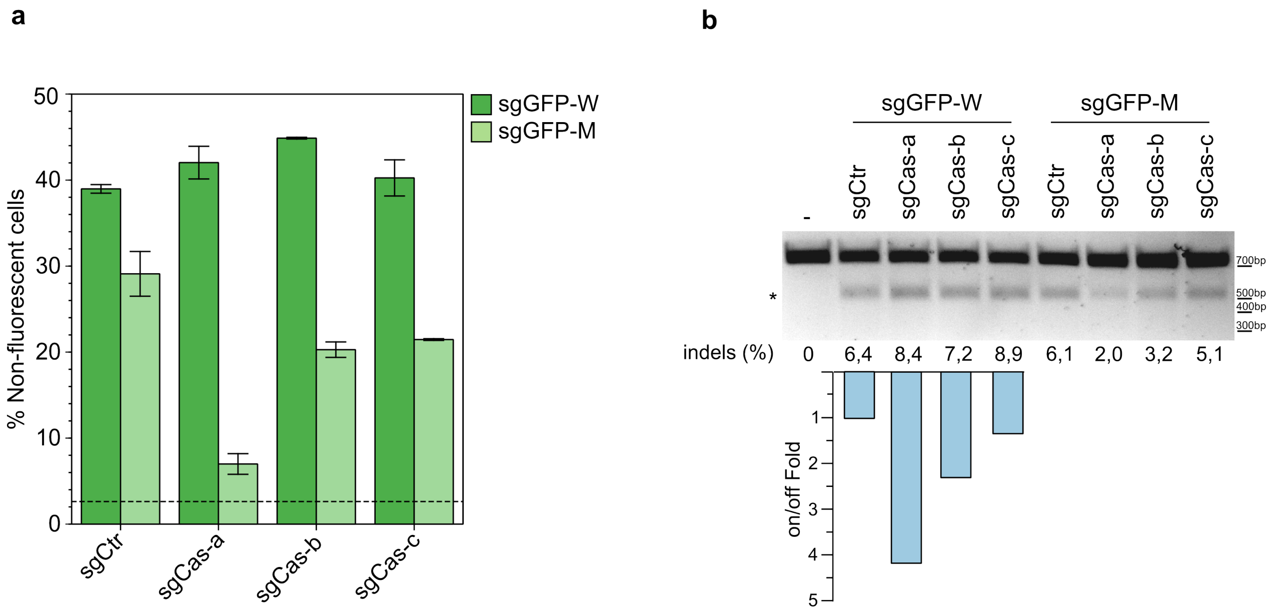

Supplementary Figure 3. EGFP disruption by SLiCES circuits. (**a**) Percentage of non-fluorescent 293-iEGFP cells obtained after expression of different self-limiting SpCas9 circuits. Cells were transfected with sgRNAs perfectly matching (sgGFP-W) or containing one mismatch (sgGFP-M) with the EGFP ORF together with three sgRNAs targeting the SpCas9 ORF (sgCas-a, -b, -c) or a control sgRNA (sgCtr), as indicated. The dashed line represents the average background of EGFP negative cells. Error bars represent s.e.m. for n=2. Data presented as mean ± s.e.m. for n=2 independent experiments. **(b)** Representative T7 Endonuclease assay from cells expressing different SLiCES circuits. The on/off specificity ratio was calculated by measuring indel formation in the EGFP gene in the presence of sgGFP-W or sgGFP-M together with a control sgRNA or the three sgRNAs targeting the SpCas9 ORF (sgCas-a, -b, -c). Lane (-) corresponds to a reference sample containing the non-targeting sgCtr only. (*) Indicates the expected band obtained by T7 endonuclease activity.

Supplementary Figure 4

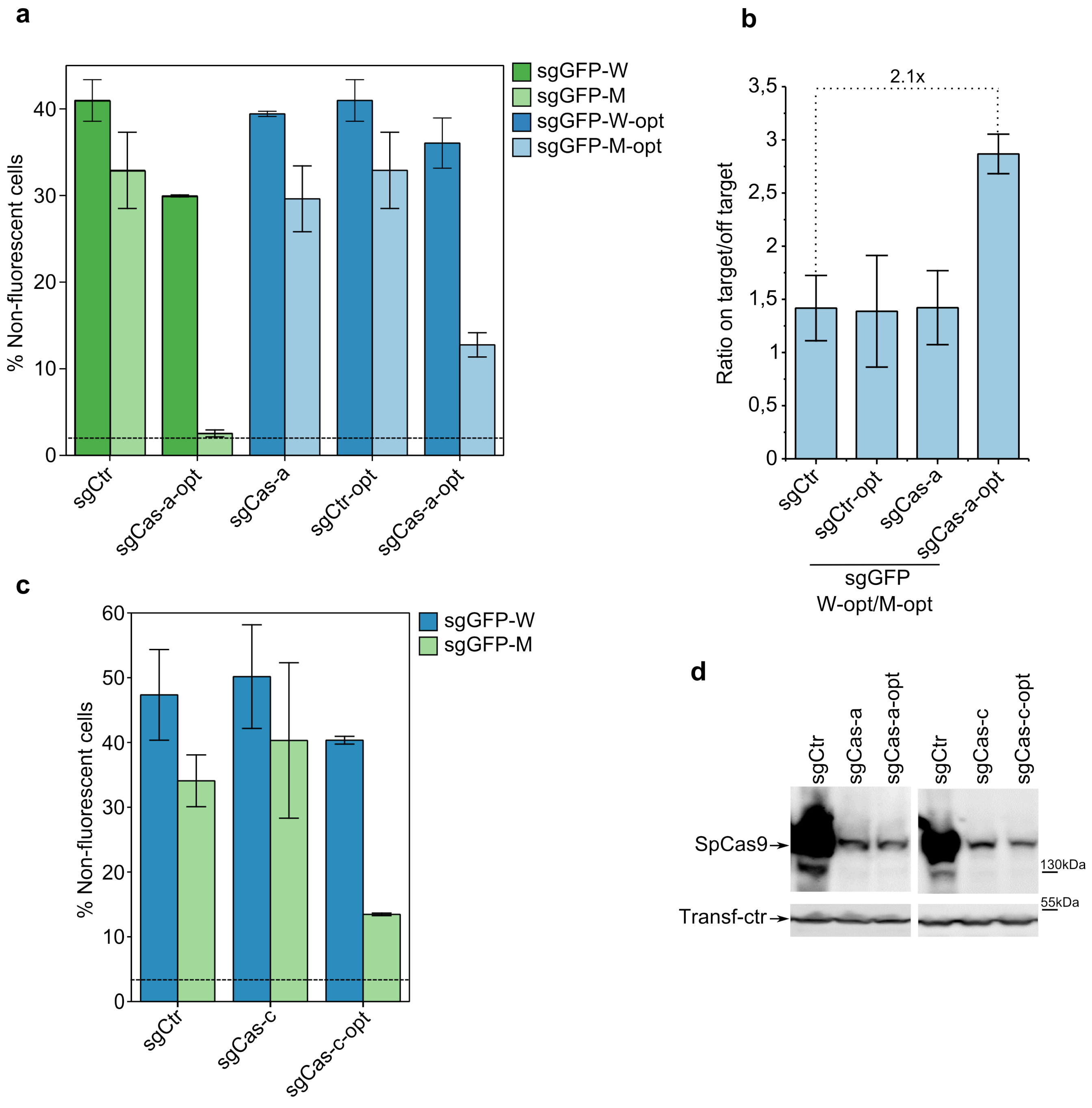

Supplementary Figure 4. (**a**) Percentage of non-fluorescent 293-iEGFP cells obtained after transfection of SpCas9 with sgRNAs targeting EGFP (sgGFP-W or sgGFP-W-opt, if optimized) or containing a single mismatch (sgGFP-M or sgGFP-M-opt, if optimized) together with the sgCas-a. The optimized version of the SLiCES sgRNA (sgCas-a-opt) was tested with both standard and optimized sgRNAs targeting EGFP, as indicated. Data presented as mean $\pm$ s.e.m. for n=2 independent experiments. (**b**) Target specificity of SpCas9 activity from dataset in (**a**) expressed as on/off ratios. Dotted lines indicate fold improvement specificity of the relative histogram bars. Data presented as mean $\pm$ s.e.m. for n=2 independent experiments. (**c**) Percentage of non-fluorescent 293-iEGFP cells obtained after transfection of SpCas9 with sgRNAs targeting EGFP (sgGFP-W) or containing a single mismatch (sgGFP-M) together with sgCas-c or sgCas-c-opt, if optimized. Data presented as mean $\pm$ s.e.m. for n=2 independent experiments. (**d**) Western blot analysis of 293T cells co-transfected with SpCas9 and sgCas9-a or sgCas-a-opt and sgCas9-c or sgCas-c-opt. SpCas9 was detected using an anti-FLAG antibody. Transfection efficiency was normalized using roTag tagged MHC-Iα expression plasmid (Transf-ctr).

Supplementary Figure 5

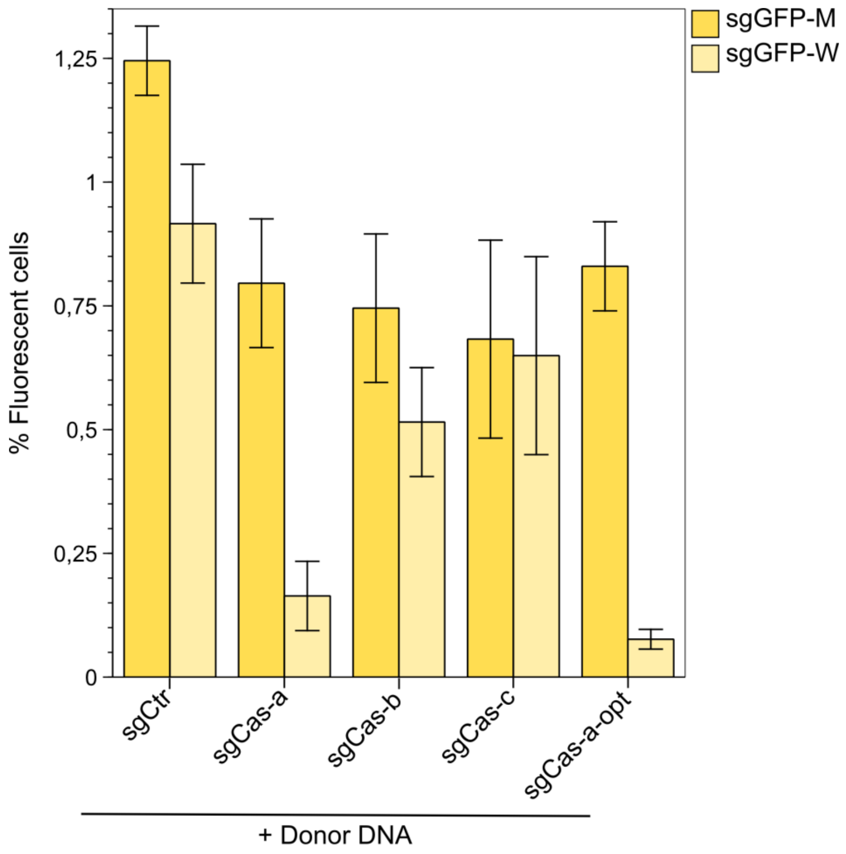

Supplementary Figure 5. Specificity of homology-directed repair mediated by SLiCES. Percentage of fluorescent 293-iY66S cells obtained after transfection with a donor DNA plasmid (carrying a non-fluorescent fragment of wt-EGFP), SpCas9 together with sgRNAs matching (sgGFP-M) or containing one mismatch with the EGFP-Y66S target sequence (sgGFP-W) and the three sgRNAs targeting the SpCas9 ORF (sgCas-a, -b, -c or sgCas-a-opt) or a control sgRNA (sgCtr), as indicated. Data presented as mean ± s.e.m. for n=2 independent experiments. Homology-directed repair in the absence of sgGFP-M or sgGFP-W was about 0.01%.

Supplementary Figure 6

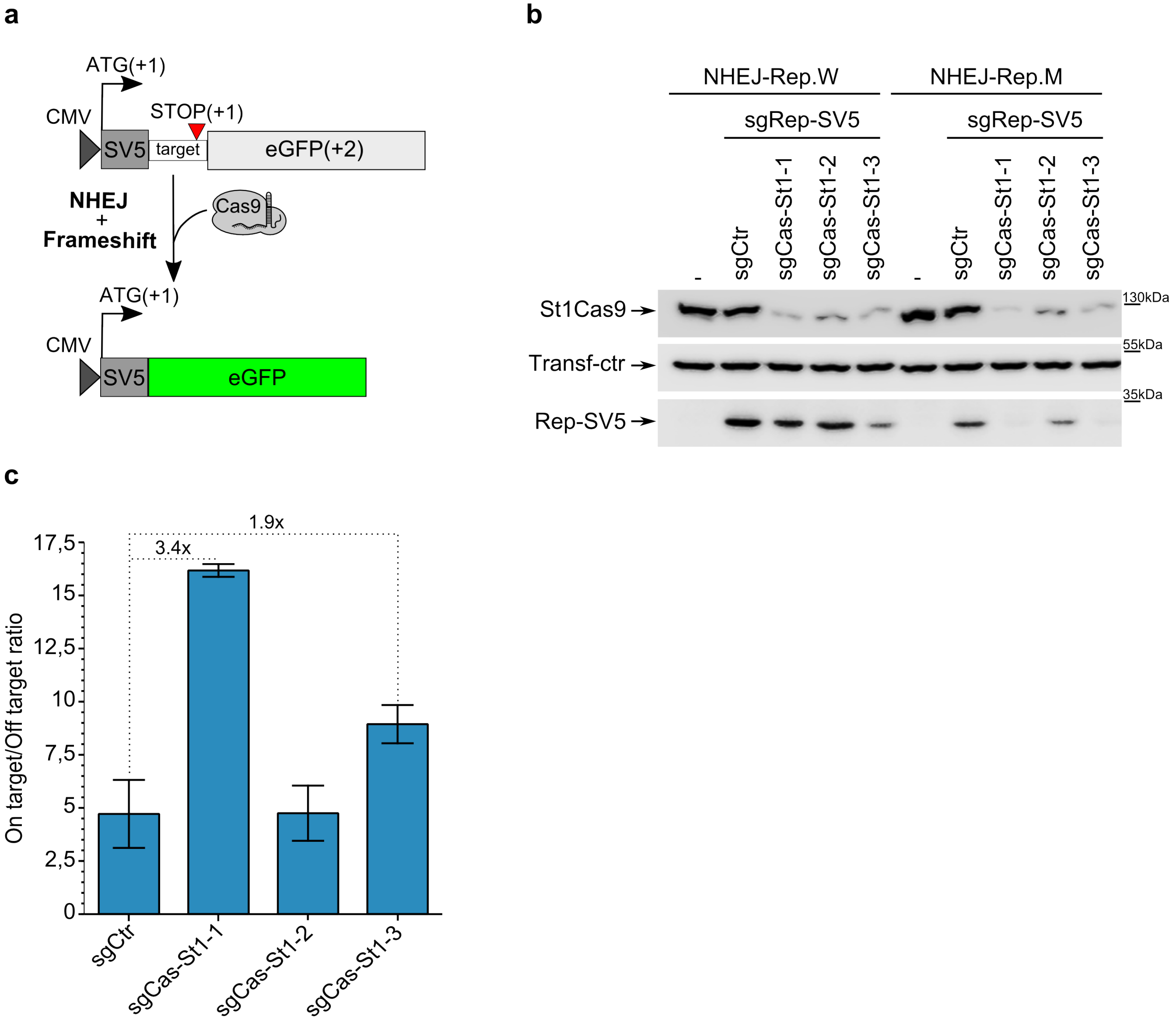

Supplementary Figure 6. Activity of SLiCES with *Streptococcus thermophilus* CRISPR1-Cas9. (**a**) Schematic representation of the SV5-GFP-based NHEJ reporter. The target sequence recognized by the sgRNA of interest is inserted between the SV5 tag and the

EGFP coding sequences, with the EGFP ORF positioned out of frame with respect to the starting ATG codon for the SV5 tag ORF. A stop codon has been added to the SV5 frame, immediately after the target sequence, to stop its translation. After SpCas9-mediated cleavage of the target sequence and repair by NHEJ, indel mutations are inserted randomly at the breakpoint, allowing the shift of the EGFP ORF in the same frame of the SV5 tag ORF. The expression of the SV5-EGFP is analyzed by fluorescence detection or by western blot analysis. (**b**) Evaluation of St1Cas9 activity expressed through the SLiCES system. Western blot of 293T cells transfected with St1Cas9, the NHEJ reporter carrying either a target sequence that fully base pairs with the sgRep-SV5 (NHEJ-Rep.W) or including one mismatch (NHEJ-Rep.M), the sgRNA sgRep-SV5 and three different St1Cas9 targeting sgRNAs (sgCas-St1, -2, -3). St1Cas9 mediated cleavages are detected by frameshift of the EGFP ORF and SV5-EGFP expression by the NHEJ reporter as described in (**a**). Lane (sgCtr) corresponds to a sample transfected with a non-self-targeting sgRNAs; lane (-) corresponds to a sample transfected with a non-targeting sgRNA. St1Cas9 was detected using an anti-FLAG antibody. Western blot is representative of n=2 independent experiments. (**c**) Modulation of St1Cas9 expression by self-limiting circuits increases on target specificity. On/off-target ratios calculated from levels of SV5-EGFP expression obtained from cells transfected with NHEJ-Rep.W or NHEJ-Rep.M together with sgRep-SV5 in combination with St1Cas9 targeting sgRNAs (sgCas-St1, -2, -3) or a non-self-targeting sgRNAs sgCtr as in (**b**). Dotted lines indicate fold improvement specificity of the relative histogram bars. Data presented as mean $\pm$ s.e.m. for n=2 independent experiments.

Supplementary Figure 7

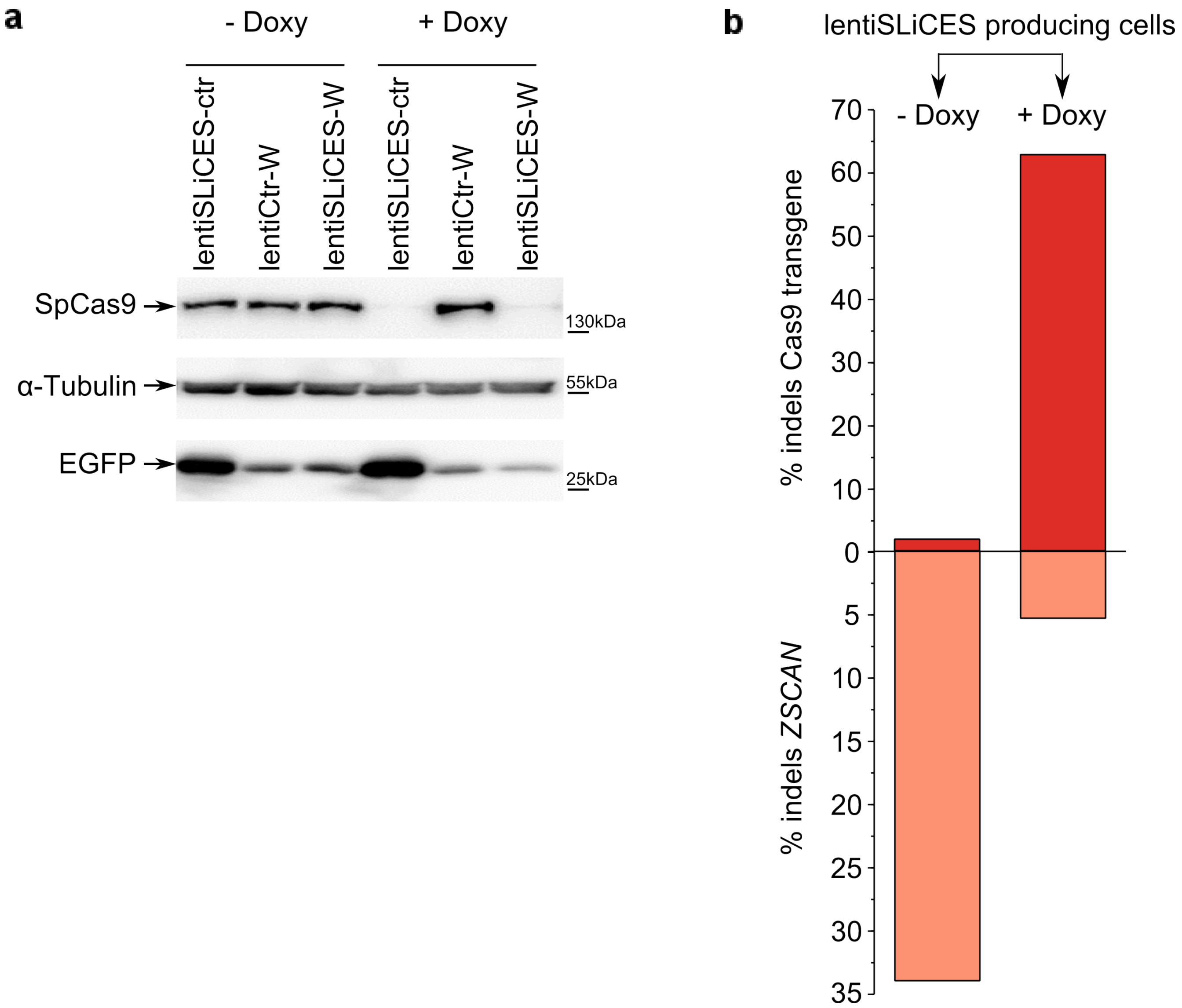

Supplementary Figure 7. lentiSLiCES circuit behaviour in viral vector packaging cells. (**a**) Western blot analysis of 293TR cells transfected with EGFP and self-limiting or non-self-limiting transfer vectors carrying sgGFP-W (lentiSLiCES-W or lentiCtr-W, respectively) or with lentiSLiCES carrying a non-targeting sgRNA (lentiSLiCES-Ctr). Cultures were treated as

indicated with doxycycline to upregulate expression of SpCas9 and of the self-targeting sgCas-a-opt. SpCas9 was detected using an anti-FLAG antibody. Western blot is representative of n=2 independent experiments. (**b**) TIDE analysis of the indels identified in the Cas9 transgene carried by the lentiSLiCES viral vectors produced in packaging cells either treated (+ Doxy) or not (- Doxy) with doxycycline (upper graph). The same viral vectors (+/- Doxy) were then used to transduce HEK293T cells and their respective editing activity towards the *ZSCAN* locus is shown as the percentage of indels detected by TIDE analysis (lower graph).

Supplementary Figure 8

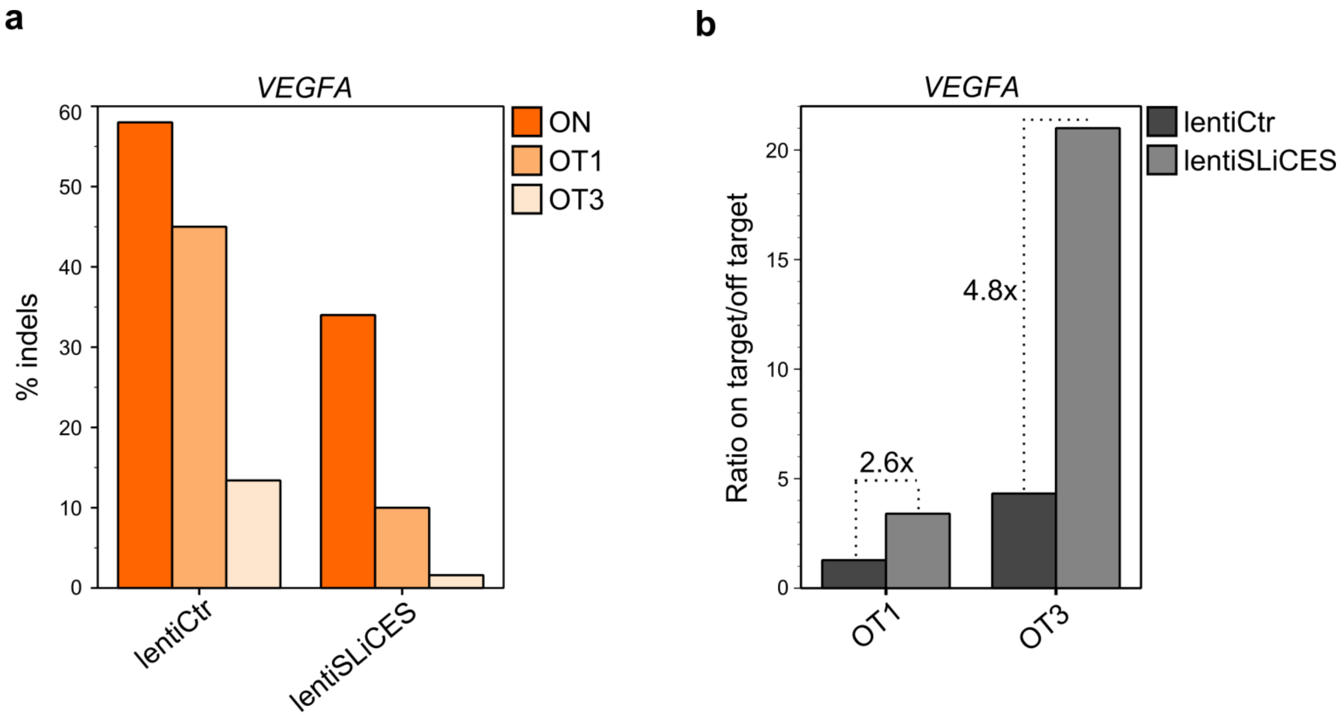

Supplementary Figure 8. Target specificity of lentiSLiCES in human neural progenitor cells. (**a**) Indels formation at the *VEGFA* locus and two previously validated off-target sites (OT1 and OT3) after long-term transduction of neural progenitor cells with lentiSLiCES or with the non-self limiting lentiCtr vectors. Editing was measured by TIDE at 30 days post-transduction and selection with blasticidin. (**b**) Target specificity of lentiSLiCES and lentiCtr in neural progenitor cells expressed as on/off-target ratios calculated from the data in (**a**). Dotted lines indicate the specificity fold increase calculated by dividing the on/off ratios of lentiSLiCES and lentiCtrl for each off-target site. Data reported a representative single experiment.

Supplementary Figure 9

ATGGGCAAGCCTATCCCCAACCCTCTCCTCGGTCTCGATTCTACGGC**TAG**CCTCGTGACCACCCTGACCTCCGGAGTGTAGGAGGAGGA
GGAGGCAGCGGCGGAGGCGGAAGCGGGCGGCCGCTAGTGAGCAAGGGCGAGGAGCTGTTCACCGGGGTGGTGCCCATCCTGGTCGAG
CTGGACGGCGACGTAAACGGCCACAAGTTCAGCGTGTCCGGCGAGGGCGAGGGCGATGCCACCTACGGCAAGCTGACCCTGAAGTTCAT
CTGCACCAC**a**GG**a**AA**a**CT**c**CC**t**GT**c**CC**t**TGGCC**a**AC**t**CT**g**GT**c**AC**t**AC**a**CT**t**AC**a**T**a**CGGCGTGCAGTGCTTCAGCCGCTACCCCGACCACA
TGAAGCAGCACGACTTCTTCAAGTCCGCCATGCCCGAAGGCTACGTCCAGGAGCGCACCATCTTCTTCAAGGACGACGGCAACTACAAGA
CCCGCGCCGAGGTGAAGTTCGAGGGCGACACCCTGGTGAACCGCATCGAGCTGAAGGGCATCGACTTCAAGGAGGACGGCAACATCCTG
GGGCACAAGCTGGAGTACAACTACAACAGCCACAACGTCTATATCATGGCCGACAAGCAGAAGAACGGCATCAAGGTGAACTTCAAGATC
CGCCACAACATCGAGGACGGCAGCGTGCAGCTCGCCGACCACTACCAGCAGAACACCCCCATCGGCGACGGCCCCGTGCTGCTGCCCGA
CAACCACTACCTGAGCACCCAGTCCGCCCTGAGCAAAGACCCCAACGAGAAGCGCGATCACATGGTCCTGCTGGAGTTCGTGACCGCCGC
CGGGATCACTCTCGGCATGGACGAGCTGTACAAATAA

Supplementary Figure 9. Rep. SV5-EGFP sequence. Coding sequence of the Cas9 activity reporter employed in this study (see Fig. 4 and Supplementary Fig. 6). The SV5 tag is indicated in blue, the desired CRISPR nuclease target sequence in gray, the linker sequence in yellow and the resistant EGFP coding sequence in green. This EGFP CDS, resistant to specific EGFP-targeting sgRNAs (sgGFP-W, -M), was obtained by introducing the synonymous mutations indicated in lowercase red to allow the insertion of corresponding target sequences into the reporter target region. The NheI and BspEI restriction sites used to clone the CRISPR nuclease target sequence are underlined, while an in frame stop codon to block translation in the absence of editing is indicated in bold.

Supplementary Figure 10

ATGGTGAGCAAGGGCGAGGAGCTGTTCACCGGGGTGGTGCCCATCCTGGTCGAGCTGGACGGCGACGTAAACGGCCACAAGTTCAGCG
TGTCCGGCGAGGGCGAGGGCGATGCCACCTACGGCAAGCTGACCCTGAAGTTCATCTGCACCAC**a**GG**a**AA**a**CT**c**CC**t**GT**c**CC**t**TGGCC**a**A
C**t**CT**g**GT**c**AC**t**AC**a**CT**t**AC**a**T**a**CGGCGTGCAGTGCTTCAGCCGCTACCCCGACCACATGAAGCAGCACGACTTCTTCAAGTCCGCCATGCC
CGAAGGCTACGTCCAGGAGCGCACCATCTTCTTCAAGGACGACGGCAACTACAAGACCCGCGCCGAGGTGAAGTTCGAGGGCGACACCC
TGGTGAACCGCATCGAGCTGAAGGGCATCGACTTCAAGGAGGACGGCAACATCCTGGGGCACAAGCTGGAGTACAACTACAACAGCCAC
AACGTCTATATCATGGCCGACAAGCAGAAGAACGGCATCAAGGTGAACTTCAAGATCCGCCACAACATCGAGGACGGCAGCGTGCAGCTC
GCCGACCACTACCAGCAGAACACCCCCATCGGCGACGGCCCCGTGCTGCTGCCCGACAACCACTACCTGAGCAAGTAA

Supplementary Figure 10. rEGFP(1-T203K-stop) donor sequence. To generated the pcDNA5-FRT-TO-rEGFP(1-T203K-stop) plasmid, synonymous substitutions were employed to prevent sgRNA retargeting after homologous recombination (highlighted in red). The key nucleotide change to restore EGFP fluorescence by reverting the Y66S mutation is underlined. The end of the 410bp 3′-homology arm (corresponding to T203K-stop) is labeled in black.

Supplementary Figure 11

TTAATGTAGTCTTATGCAATACTCTTGTAGTCTTGCAACATGGTAACGATGAGTTAGCAACATGCCTTACAAGGAGAGAAAAAGCACCGT
GCATGCCGATTGGTGGAAGTAAGGTGGTACGATCGTGCCTTATTAGGAAGGCAACAGACGGGTCTGACATGGATTGGACGAACCACTGA
ATTGCCGCATTGCAGAGATATTGTATTTAAGTGCCTAGCTCGATACATAAACGGGTCTCTCTGGTTAGACCAGATCTGAGCCTGGGAGCT
CTCTGGCTAACTAGGGAACCCACTGCTTAAGCCTCAATAAAGCTTGCCTTGAGTGCTTCAAGTAGTGTGTGCCCGTCTGTTGTGTGACTC
TGGTAACTAGAGATCCCTCAGACCCTTTTAGTCAGTGTGGAAAATCTCTAGCAGTGGCGCCCGAACAGGGACTTGAAAGCGAAAGGGAAA
CCAGAGGAGCTCTCTCGACGCAGGACTCGGCTTGCTGAAGCGCGCACGGCAAGAGGCGAGGGGCGGCGACTGGTGAGTACGCCAAAAA
TTTTGACTAGCGGAGGCTAGAAGGAGAGAGATGGGTGCGAGAGCGTCAGTATTAAGCGGGGGAGAATTAGATCGCGATGGGAAAAAAT
TCGGTTAAGGCCAGGGGGAAAGAAAAAATATAAATTAAAACATATAGTATGGGCAAGCAGGGAGCTAGAACGATTCGCAGTTAATCCTGG
CCTGTTAGAAACATCAGAAGGCTGTAGACAAATACTGGGACAGCTACAACCATCCCTTCAGACAGGATCAGAAGAACTTAGATCATTATA
TAATACAGTAGCAACCCTCTATTGTGTGCATCAAAGGATAGAGATAAAAGACACCAAGGAAGCTTTAGACAAGATAGAGGAAGAGCAAAA
CAAAAGTAAGACCACCGCACAGCAAGCGGCCGCTGATCTTCAGACCTGGAGGAGGAGATATGAGGGACAATTGGAGAAGTGAATTATAT
AAATATAAAGTAGTAAAAATTGAACCATTAGGAGTAGCACCCACCAAGGCAAAGAGAAGAGTGGTGCAGAGAGAAAAAAGAGCAGTGGG
AATAGGAGCTTTGTTCCTTGGGTTCTTGGGAGCAGCAGGAAGCACTATGGGCGCAGCGTCAATGACGCTGACGGTACAGGCCAGACAAT
TATTGTCTGGTATAGTGCAGCAGCAGAACAATTTGCTGAGGGCTATTGAGGCGCAACAGCATCTGTTGCAACTCACAGTCTGGGGCATCA
AGCAGCTCCAGGCAAGAATCCTGGCTGTGGAAAGATACCTAAAGGATCAACAGCTCCTGGGGATTTGGGGTTGCTCTGGAAAACTCATTT
GCACCACTGCTGTGCCTTGGAATGCTAGTTGGAGTAATAAATCTCTGGAACAGATTTGGAATCACACGACCTGGATGGAGTGGGACAGA
GAAATTAACAATTACACAAGCTTAATACACTCCTTAATTGAAGAATCGCAAAACCAGCAAGAAAAGAATGAACAAGAATTATTGGAATTAG
ATAAATGGGCAAGTTTGTGGAATTGGTTTAACATAACAAATTGGCTGTGGTATATAAAATTATTCATAATGATAGTAGGAGGCTTGGTAG
GTTTAAGAATAGTTTTTGCTGTACTTTCTATAGTGAATAGAGTTAGGCAGGGATATTCACCATTATCGTTTCAGACCCACCTCCCAACCCC
GAGGGGACCCAGAGAGGGCCTATTTCCCATGATTCCTTCATATTTGCATATACGATACAAGGCTGTTAGAGAGATAATTAGAATTAATTT
GACTGTAAACACAAAGATATTAGTACAAAATACGTGACGTAGAAAGTAATAATTTCTTGGGTAGTTTGCAGTTTTAAAATTATGTTTTAAA
ATGGACTATCATATGCTTACCGTAACTTGAAAGTATTTCGATTTCTTGGCTTTATATATCTTGTGGAAAGGACGAAACACCGGAGACGGT
TGTAAATGAGCACACAAAATACACATGCTAAAATATTATATTCTATGACCTTTATAAAATCAACCAAAATCTTCTTTTTAATAACTTTAGTA
TCAATAATTAGAATTTTTATGTTCCTTTTTGCAAACTTTTAATAAAAATGAGCAAAATAAAAAAACGCTAGTTTTAGTAACTCGCGTTGTTT
TCTTCACCTTTAATAATAGCTACTCCACCACTTGTTCCTAAGCGGTCAGCTCCTGCTTCAATCATTTTTTGAGCATCTTCAAATGTTCTAAC
TCCACCAGCTGCTTTAACTAAAGCATTGTCTTTAACAACTGACTTCATTAGTTTAACATCTTCAAATGTTGCACCTGATTTTGAAAATCCT
GTTGATGTTTTAACAAATTCTAATCCAGCTTCAACAGCTATTTCACAAGCTTTCATGATTTCTTCTTTTGTTAATAAACAATTTTCCATAAT
ACATTTAACAACATGTGATCCAGCTGCTTTTTTTACAGCTTTCATGTCTTCTAAAACTAATTCATAATTTTTGTCTTTTAATGCACCAATAT
TTAATACCATATCAATTTCTGTTGCACCATCTTTAATTGCTTCAGAAACTTCGAATGCTTTTGTAGCTGTTGTGCATGCACCTAGAGGAAA
ACCTACAACATTTGTTATTCCTACATTTGTGCCTTTTAATAATTCTTTACAATAGCTTGTTCAATATGAATTAACACAAACTGTTGCAAAAT
CAAATTCAATTGCTTCATCACATAATTGTTTAATTTCAGCTTTCGTAGCATCTTGTTTTAATAATGTGTGATCTATATATTTGTTTAGTTTC
ATTTTTTCTCCTATATATTCATTTTTAATTTTAATTCTTTAATAATTTCGTCTACTTTAACTTTAGCGTTTTGAACAGATTCACCAACACCT
ATAAAATAAATTTTTAGTTTAGGTTCAGTTCCACTTGGGCGAACAGCAAATCATGACTTATCTTCTAAATAAAATTTTAGTAAGTCTTGTC
CTGGCATATTATACATTCCATCGATGTAGTCTTCAACATTAACAACTTTAAGTCCAGCAATTTGAGTTAAGGGTGTTGCTCTCAATGATTT
CATTAATGGTTCAATTTTTAATTTCTTTTCTTCTGGTTTAAAATTCAAGTTTAAAGTGAAAGTGTAATATGCACCCATTTCTTTAAATAAAT
CTTCTAAATAGTCTACTAATGTTTTATTTTGTTTTTTATAAAATCAAGCAGCCTCTGCTATTAATATAGAAGCTTGTATTCCATCTTTATCT
CTAGCTGAGTCATCAATTACATATCCATAACTTTCTTCATAAGCAAAAACAAAATTTAATCCGTTATCTTCTTCTTTAGCAATTTCTCTACC
CATTCATTTAAATCCAGTTAAAGTTTTTACAATATTAACTCCATATTTTTCATGAGCGATTCTATCACCCAAATCACTTGTTACAAAACTTG
AATATAGAGCCGGATTTTTTGGAATGCTATTTAAGCGTTTTAGATTTGATAATTTTCAATCAATTAAAATTGGTCCTGTTTGATTTCCATC
TAATCTTACAAAATGACCATCATGTTTTATTGCCATTCCAAATCTGTCAGCATCTGGGTCATTCATAATAATAATATCTGCATCATGTTTAA
TACCATATTCAAGCGGTATTTTTCATGCAGGATCAAATTCTGGATTTGGATTTACAACATTTTTAAATGTTTCATCTTCAAATGCATGCTC
TTCAACCTCAATAACGTTATATCCTGATTCACGTAATATTTTTGGGGTAAATTTAGTTCCTGTTCCATTAACTGCGCTAAAAATAATTTTTA
AATCTTTTTTAGCTTCTTGCTCTTTTTTGTACGTCTCTGTTTTAGAGCTAGAAATAGCAAGTTAAAATAAGGCTAGTCCGTTATCAACTTG
AAAAAGTGGCACCGAGTCGGTGCTTTTTTGaattctagtagaattgaggtaccAATATTTGCATGTCGCTATGTGTTCTGGGAAATCACCATAAAC
GTGAAATCCCTATCAGtGATAGAGACTTATAAGTTCCCTATCAGTGATAGAGACACCgTACGCCGGCTACATTGACGGGTTTAAGAGCTAT
GCTGGAAACAGCATAGCAAGTTTAAATAAGGCTAGTCCGTTATCAACTTGAAAAAGTGGCACCGAGTCGGTGCTTTTTTcAATTCTAGATC
TTGAGACAAATGGCAGTATTCATCCACAATTTTAAAAGAAAAGGGGGGATTGGGGGGTACAGTGCAGGGGAAAGAATAGTAGACATAAT
AGCAACAGACATACAAACTAAAGAATTACAAAAACAAATTACAAAAATTCAAAATTTTCGGGTTTATTACAGGGACAGCAGAGATCCACTT
TGGCGCCGGCtcgagGTTGACATTGATTATTGACTAGTTATTAATAGTAATCAATTACGGGGTCATTAGTTCATAGCCCATATATGGAGTT
CCGCGTTACATAACTTACGGTAAATGGCCCGCCTGGCTGACCGCCCAACGACCCCCGCCCATTGACGTCAATAATGACGTATGTTCCCAT
AGTAACGCCAATAGGGACTTTCCATTGACGTCAATGGGTGGAGTATTTACGGTAAACTGCCCACTTGGCAGTACATCAAGTGTATCATAT
GCCAAGTACGCCCCCTATTGACGTCAATGACGGTAAATGGCCCGCCTGGCATTATGCCCAGTACATGACCTTATGGGACTTTCCTACTTG
GCAGTACATCTACGTATTAGTCATCGCTATTACCATGGTGATGCGGTTTTGGCAGTACATCAATGGGCGTGGATAGCGGTTTGACTCACG
GGGATTTCCAAGTCTCCACCCCATTGACGTCAATGGGAGTTTGTTTTGGCACCAAAATCAACGGGACTTTCCAAAATGTCGTAACAACTC
CGCCCCATTGACGCAAATGGGCGGTAGGCGTGTACGGTGGGAGGTCTATATAAGCAGAGCTCTCCCTATCAGTGATAGAGATCTCCCTA
TCAGTGATAGAGATCGTCGACGAGCTCGTTTAGTGAACCGTCAGATCGCCTGGAGAggatcCGCCACCATGGATTACAAAGACGATGACGA

TAAGATGGCCCCAAAGAAGAAGCGGAAGGTCGGTATCCACGGAGTCCCAGCAGCCGACAAGAAGTACAGCATCGGCCTGGACATCGGCA
CCAACTCTGTGGGCTGGGCCGTGATCACCGACGAGTACAAGGTGCCCAGCAAGAAATTCAAGGTGCTGGGCAACACCGACCGGCACAGC
ATCAAGAAGAACCTGATCGGAGCCCTGCTGTTCGACAGCGGCGAAACAGCCGAGGCCACCCGGCTGAAGAGAACCGCCAGAAGAAGATA
CACCAGACGGAAGAACCGGATCTGCTATCTGCAAGAGATCTTCAGCAACGAGATGGCCAAGGTGGACGACAGCTTCTTCCACAGACTGGA
AGAGTCCTTCCTGGTGGAAGAGGATAAGAAGCACGAGCGGCACCCCATCTTCGGCAACATCGTGGACGAGGTGGCCTACCACGAGAAGT
ACCCCACCATCTACCACCTGAGAAAGAAACTGGTGGACAGCACCGACAAGGCCGACCTGCGGCTGATCTATCTGGCCCTGGCCCACATGA
TCAAGTTCCGGGGCCACTTCCTGATCGAGGGCGACCTGAACCCCGACAACAGCGACGTGGACAAGCTGTTCATCCAGCTGGTGCAGACC
TACAACCAGCTGTTCGAGGAAAACCCCATCAACGCCAGCGGCGTGGACGCCAAGGCCATCCTGTCTGCCAGACTGAGCAAGAGCAGACG
GCTGGAAAATCTGATCGCCCAGCTGCCCGGCGAGAAGAAGAATGGCCTGTTCGGCAACCTGATTGCCCTGAGCCTGGGCCTGACCCCCA
ACTTCAAGAGCAACTTCGACCTGGCCGAGGATGCCAAACTGCAGCTGAGCAAGGACACCTACGACGACGACCTGGACAACCTGCTGGCCC
AGATCGGCGACCAGTACGCCGACCTGTTTCTGGCCGCCAAGAACCTGTCCGACGCCATCCTGCTGAGCGACATCCTGAGAGTGAACACCG
AGATCACCAAGGCCCCCCTGAGCGCCTCTATGATCAAGAGATACGACGAGCACCACCAGGACCTGACCCTGCTGAAAGCTCTCGTGCGGC
AGCAGCTGCCTGAGAAGTACAAAGAGATTTTCTTCGACCAGAGCAAGAACGGCTACGCCGGCTACATTGACGGCGGAGCCAGCCAGGAA
GAGTTCTACAAGTTCATCAAGCCCATCCTGGAAAAGATGGACGGCACCGAGGAACTGCTCGTGAAGCTGAACAGAGAGGACCTGCTGCG
GAAGCAGCGGACCTTCGACAACGGCAGCATCCCCCACCAGATCCACCTGGGAGAGCTGCACGCCATTCTGCGGCGGCAGGAAGATTTTT
ACCCATTCCTGAAGGACAACCGGGAAAAGATCGAGAAGATCCTGACCTTCCGCATCCCCTACTACGTGGGCCCTCTGGCCAGGGGAAACA
GCAGATTCGCCTGGATGACCAGAAAGAGCGAGGAAACCATCACCCCCTGGAACTTCGAGGAAGTGGTGGACAAGGGCGCTTCCGCCCAG
AGCTTCATCGAGCGGATGACCAACTTCGATAAGAACCTGCCCAACGAGAAGGTGCTGCCCAAGCACAGCCTGCTGTACGAGTACTTCACC
GTGTATAACGAGCTGACCAAAGTGAAATACGTGACCGAGGGAATGAGAAAGCCCGCCTTCCTGAGCGGCGAGCAGAAAAAGGCCATCGT
GGACCTGCTGTTCAAGACCAACCGGAAAGTGACCGTGAAGCAGCTGAAAGAGGACTACTTCAAGAAAATCGAGTGCTTCGACTCCGTGG
AAATCTCCGGCGTGGAAGATCGGTTCAACGCCTCCCTGGGCACATACCACGATCTGCTGAAAATTATCAAGGACAAGGACTTCCTGGACA
ATGAGGAAAACGAGGACATTCTGGAAGATATCGTGCTGACCCTGACACTGTTTGAGGACAGAGAGATGATCGAGGAACGGCTGAAAACC
TATGCCCACCTGTTCGACGACAAAGTGATGAAGCAGCTGAAGCGGCGGAGATACACCGGCTGGGGCAGGCTGAGCCGGAAGCTGATCAA
CGGCATCCGGGACAAGCAGTCCGGCAAGACAATCCTGGATTTCCTGAAGTCCGACGGCTTCGCCAACAGAAACTTCATGCAGCTGATCCA
CGACGACAGCCTGACCTTTAAAGAGGACATCCAGAAAGCCCAGGTAAGGGGCTCACAGTAGCAGGCTTGAGGTCTGGACATATATATGG
GTGACAATGACATCCACTTTGCCTTTCTCTCCACAGGTGTCCGGCCAGGGCGATAGCCTGCACGAGCACATTGCCAATCTGGCCGGCAGC
CCCGCCATTAAGAAGGGCATCCTGCAGACAGTGAAGGTGGTGGACGAGCTCGTGAAAGTGATGGGCCGGCACAAGCCCGAGAACATCGT
GATCGAAATGGCCAGAGAGAACCAGACCACCCAGAAGGGACAGAAGAACAGCCGCGAGAGAATGAAGCGGATCGAAGAGGGCATCAAAG
AGCTGGGCAGCCAGATCCTGAAAGAACACCCCGTGGAAAACACCCAGCTGCAGAACGAGAAGCTGTACCTGTACTACCTGCAGAATGGG
CGGGATATGTACGTGGACCAGGAACTGGACATCAACCGGCTGTCCGACTACGATGTGGACCATATCGTGCCTCAGAGCTTTCTGAAGGA
CGACTCCATCGACAACAAGGTGCTGACCAGAAGCGACAAGAACCGGGGCAAGAGCGACAACGTGCCCTCCGAAGAGGTCGTGAAGAAGA
TGAAGAACTACTGGCGGCAGCTGCTGAACGCCAAGCTGATTACCCAGAGAAAGTTCGACAATCTGACCAAGGCCGAGAGAGGCGGCCTG
AGCGAACTGGATAAGGCCGGCTTCATCAAGAGACAGCTGGTGGAAACCCGGCAGATCACAAAGCACGTGGCACAGATCCTGGACTCCCG
GATGAACACTAAGTACGACGAGAATGACAAGCTGATCCGGGAAGTGAAAGTGATCACCCTGAAGTCCAAGCTGGTGTCCGATTTCCGGA
AGGATTTCCAGTTTTACAAAGTGCGCGAGATCAACAACTACCACCACGCCCACGACGCCTACCTGAACGCCGTCGTGGGAACCGCCCTGA
TCAAAAAGTACCCTAAGCTGGAAAGCGAGTTCGTGTACGGCGACTACAAGGTGTACGACGTGCGGAAGATGATCGCCAAGAGCGAGCAG
GAAATCGGCAAGGCTACCGCCAAGTACTTCTTCTACAGCAACATCATGAACTTTTTCAAGACCGAGATTACCCTGGCCAACGGCGAGATC
CGGAAGCGGCCTCTGATCGAGACAAACGGCGAAACCGGGGAGATCGTGTGGGATAAGGGCCGGGATTTTGCCACCGTGCGGAAAGTGC
TGAGCATGCCCCAAGTGAATATCGTGAAAAAGACCGAGGTGCAGACAGGCGGCTTCAGCAAAGAGTCTATCCTGCCCAAGAGGAACAGC
GATAAGCTGATCGCCAGAAAGAAGGACTGGGACCCTAAGAAGTACGGCGGCTTCGACAGCCCCACCGTGGCCTATTCTGTGCTGGTGGT
GGCCAAAGTGGAAAAGGGCAAGTCCAAGAAACTGAAGAGTGTGAAAGAGCTGCTGGGGATCACCATCATGGAAAGAAGCAGCTTCGAGA
AGAATCCCATCGACTTTCTGGAAGCCAAGGGCTACAAAGAAGTGAAAAAGGACCTGATCATCAAGCTGCCTAAGTACTCCCTGTTCGAGC
TGGAAAACGGCCGGAAGAGAATGCTGGCCTCTGCCGGCGAACTGCAGAAGGGAAACGAACTGGCCCTGCCCTCCAAATATGTGAACTTC
CTGTACCTGGCCAGCCACTATGAGAAGCTGAAGGGCTCCCCCGAGGATAATGAGCAGAAACAGCTGTTTGTGGAACAGCACAAGCACTAC
CTGGACGAGATCATCGAGCAGATCAGCGAGTTCTCCAAGAGAGTGATCCTGGCCGACGCTAATCTGGACAAAGTGCTGTCCGCCTACAAC
AAGCACCGGGATAAGCCCATCAGAGAGCAGGCCGAGAATATCATCCACCTGTTTACCCTGACCAATCTGGGAGCCCCTGCCGCCTTCAAG
TACTTTGACACCACCATCGACCGGAAGAGGTACACCAGCACCAAAGAGGTGCTGGACGCCACCCTGATCCACCAGAGCATCACCGGCCTG
TACGAGACACGGATCGACCTGTCTCAGCTGGGAGGCGACAAGCGTCCTGCTGCTACTAAGAAAGCTGGTCAAGCTAAGAAAAAGAAAGct
agcTGATAATGTACACGCGTGTTATTTTCCACCATATTGCCGTCTTTTGGCAATGTGAGGGCCCGGAAACCTGGCCCTGTCTTCTTGACGA
GCATTCCTAGGGGTCTTTCCCCTCTCGCCAAAGGAATGCAAGGTCTGTTGAATGTCGTGAAGGAAGCAGTTCCTCTGGAAGCTTCTTGAA
GACAAACAACGTCTGTAGCGACCCTTTGCAGGCAGCGGAACCCCCCACCTGGCGACAGGTGCCTCTGCGGCCAAAAGCCACGTGTATAA
GATACACCTGCAAAGGCGGCACAACCCCAGTGCCACGTTGTGAGTTGGATAGTTGTGGAAAGAGTCAAATGGCTCTCCTCAAGCGTATTC
AACAAGGGGCTGAAGGATGCCCAGAAGGTACCCCATTGTATGGGATCTGATCTGGGGCCTCGGTGCACATGCTTTACATGTGTTTAGTC
GAGGTTAAAAAACGTCTAGGCCCCCCGAACCACGGGGACGTGGTTTTCCTTTGAAAAACACGATGATAACCGGTATGGCCAAGCCTTTGT
CTCAAGAAGAATCCACCCTCATTGAAAGAGCAACGGCTACAATCAACAGCATCCCCATCTCTGAAGACTACAGCGTCGCCAGCGCAGCTC
TCTCTAGCGACGGCCGCATCTTCACTGGTGTCAATGTATATCATTTTACTGGGGGACCTTGTGCAGAACTCGTGGTGCTGGGCACTGCTG
CTGCTGCGGCAGCTGGCAACCTGACTTGTATCGTCGCGATCGGAAATGAGAACAGGGGCATCTTGAGCCCCTGCGGACGGTGCCGACAG
GTGCTTCTCGATCTGCATCCTGGGATCAAAGCCATAGTGAAGGACAGTGATGGACAGCCGACGGCAGTTGGGATTCGTGAATTGCTGCC
CTCTGGTTATGTGTGGGAGGGCTAAACGCGTTAAGTCGACAATCAACCTCTGGATTACAAAATTTGTGAAAGATTGACTGGTATTCTTAA
CTATGTTGCTCCTTTTACGCTATGTGGATACGCTGCTTTAATGCCTTTGTATCATGCTATTGCTTCCCGTATGGCTTTCATTTTCTCCTCC
TTGTATAAATCCTGGTTGCTGTCTCTTTATGAGGAGTTGTGGCCCGTTGTCAGGCAACGTGGCGTGGTGTGCACTGTGTTTGCTGACGC

AACCCCCACTGGTTGGGGCATTGCCACCACCTGTCAGCTCCTTTCCGGGACTTTCGCTTTCCCCCTCCCTATTGCCACGGCGGAACTCAT
CGCCGCCTGCCTTGCCCGCTGCTGGACAGGGGCTCGGCTGTTGGGCACTGACAATTCCGTGGTGTTGTCGGGGAAATCATCGTCCTTTC
CTTGGCTGCTCGCCTGTGTTGCCACCTGGATTCTGCGCGGGACGTCCTTCTGCTACGTCCCTTCGGCCCTCAATCCAGCGGACCTTCCTT
CCCGCGGCCTGCTGCCGGCTCTGCGGCCTCTTCCGCGTCTTCGCCTTCGCCCTCAGACGAGTCGGATCTCCCTTTGGGCCGCCTCCCCGC
GTCGACTTTAAGACCAATGACTTACAAGGCAGCTGTAGATCTTAGCCACTTTTTAAAAGAAAAGGGGGGACTGGAAGGGCTAATTCACTC
CCAACGAAGACAAGATCTGCTTTTTGCTTGTACTGGGTCTCTCTGGTTAGACCAGATCTGAGCCTGGGAGCTCTCTGGCTAACTAGGGAA
CCCACTGCTTAAGCCTCAATAAAGCTTGCCTTGAGTGCTTCAAGTAGTGTGTGCCCGTCTGTTGTGTGACTCTGGTAACTAGAGATCCCT
CAGACCCTTTTAGTCAGTGTGGAAAATCTCTAGCAG

5′LTR, 3′LTR-SIN, hU6 promoter, gRNA backbone, stuffer fragment, hH1TetO promoter, Cas-a spacer sequence, optimized gRNA backbone, CMV-TetO promoter, FLAG-NLS-SpCas9-NLS, intron, ECMV-IRES, blasticidin resistance gene, WPRE.

Supplementary Figure 11. lentiSLiCES transfer vector sequence.

Supplementary Figure 12

a

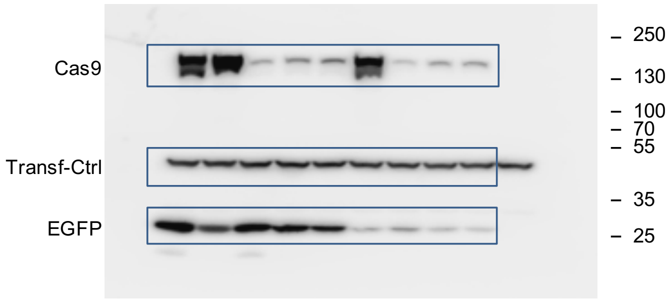

b

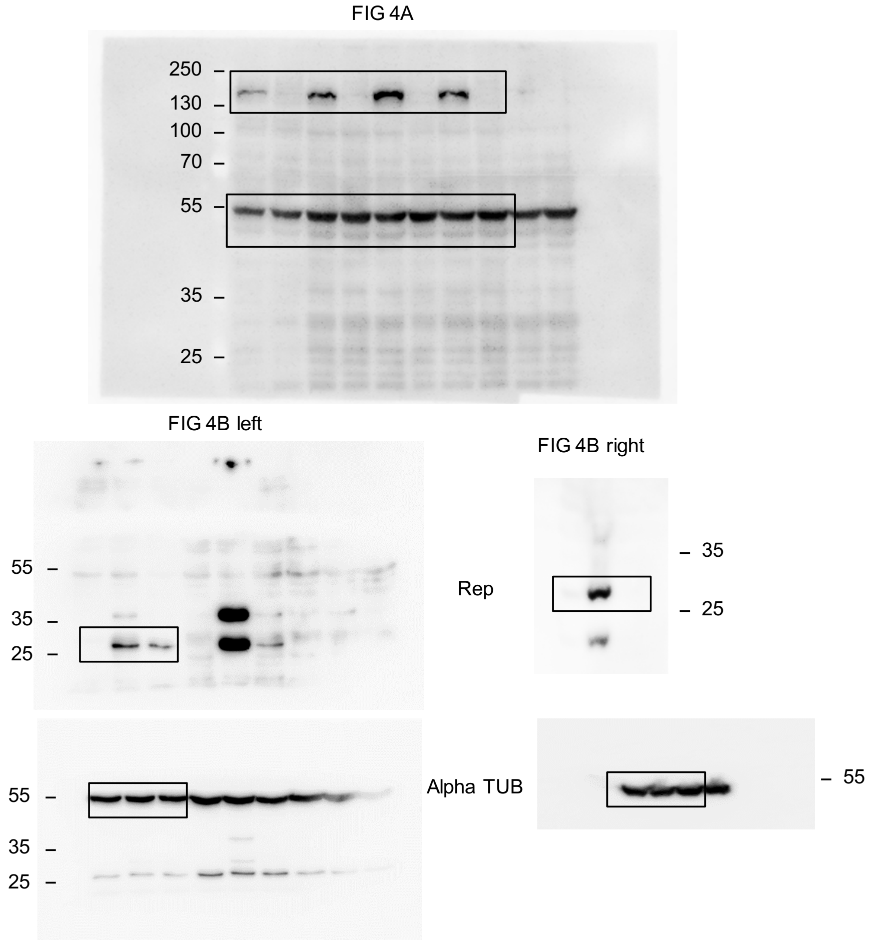

c

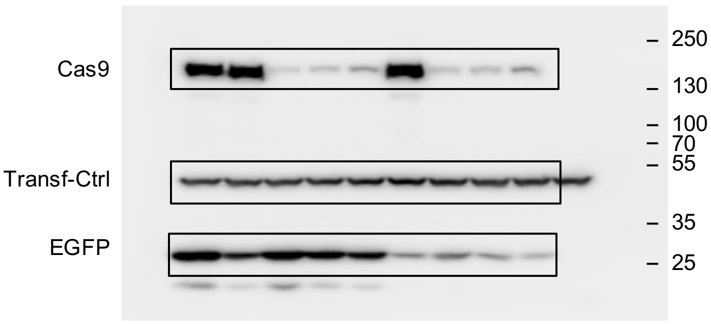
Cas9
Transf-Ctrl
EGFP
250
130
100
70
55
35
25

d

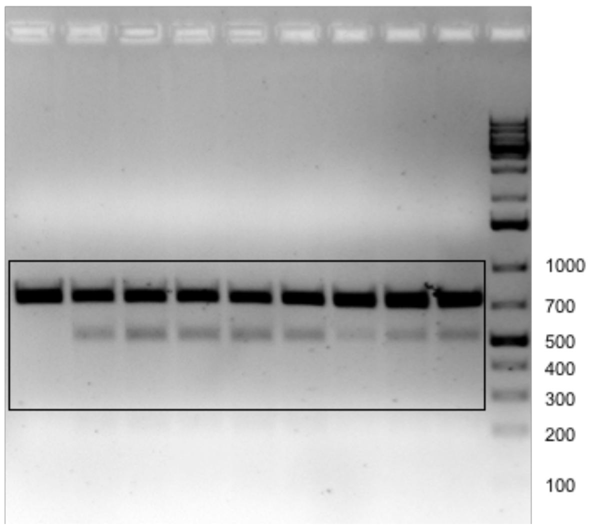
1000
700
500
400
300
200
100

e

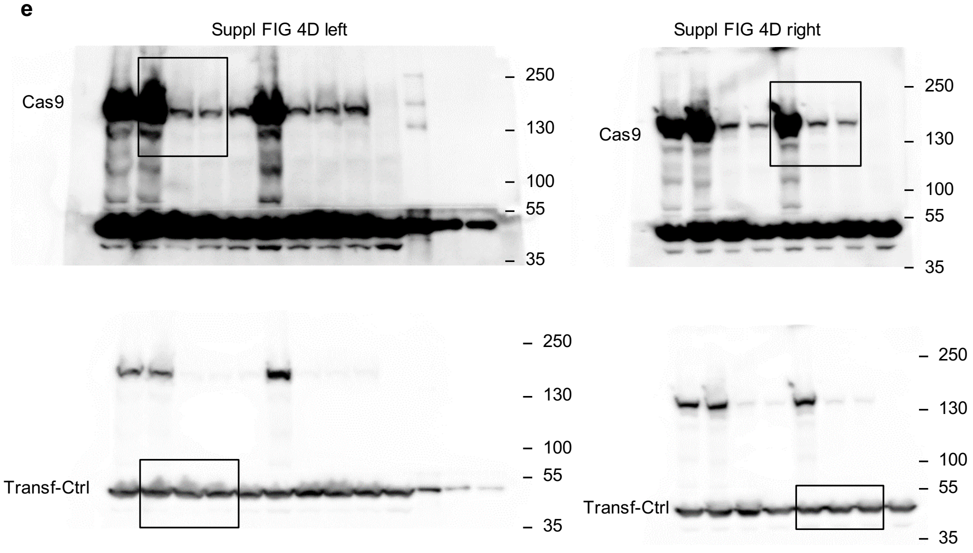
Suppl FIG 4D left
Suppl FIG 4D right
Cas9
Transf-Ctrl
250
130
100
55
35

f

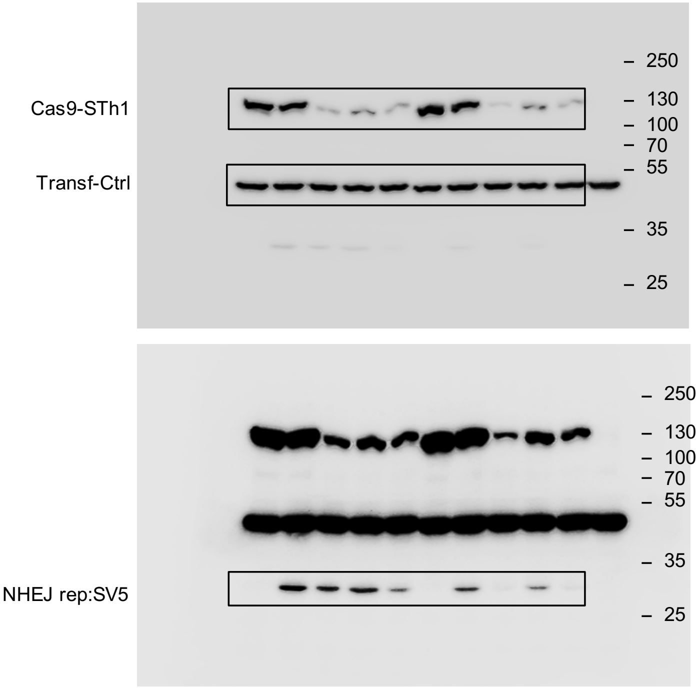

Cas9-STh1
Transf-Ctrl
NHEJ rep:SV5
250
130
100
70
55
35
25

g

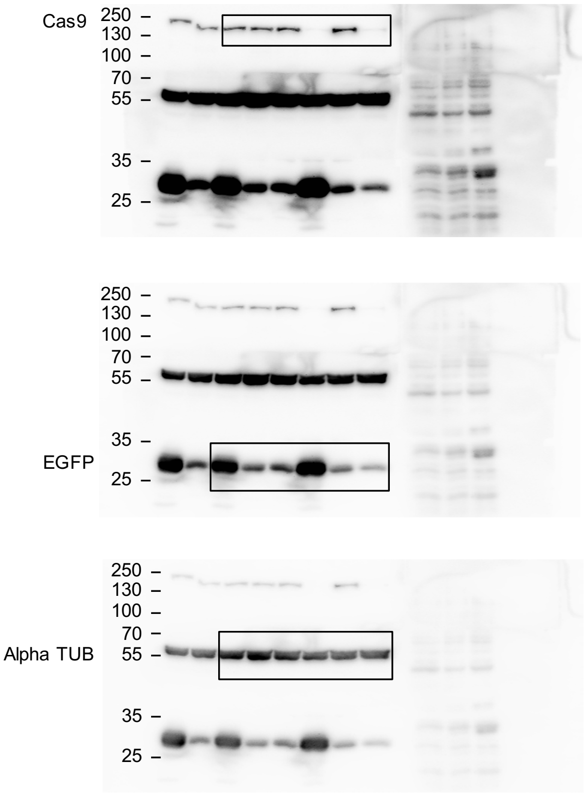

Cas9
EGFP
Alpha TUB
250
130
100
70
55
35
25

Supplementary Figure 12. (**a**) Uncropped immunoblots for Fig. 1c. The black squares indicate the sections shown in Fig. 1c. (**b**) Uncropped immunoblots for Fig. 4a-b. The black squares indicate the sections shown in Fig. 4a-b. (**c**) Uncropped immunoblots for Supplementary Fig. 2b. The black squares indicate the sections shown in Supplementary Fig. 2b. (**d**) Uncropped agarose gel image for Supplementary Fig. 3b. The black square indicates the section shown in Supplementary Fig. 3b. (**e**) Uncropped immunoblots for Supplementary Fig. 4d. The black squares indicate the sections shown in Supplementary Fig. 4d. (**f**) Uncropped immunoblots for Supplementary Fig. 6b. The black squares indicate the sections shown in Supplementary Fig. 6b. (**g**) Uncropped immunoblots for Supplementary Fig. 7a. The black squares indicate the sections shown in Supplementary Fig. 7a.

Supplementary Table 1. Summary of potential mismatched sites in the reference human genome for the self-targeting sgRNAs.

sRNA	Target sequence	mismatches to on-target site*						
		1	2	3	4	5	6	Total
Cas-a	TACGCCGGCTACATTGACGGCGG	0	0	0	13	214	2989	3216
Cas-b	GATCCTTGTAGTCTCCGTCGTGG	0	0	0	22	633	6710	7365
Cas-c	GGCTACGCCGGCTACATTGACGG	0	0	3	28	497	5157	5685

*Determined using Cas-OFFinder (http://www.rgenome.net/cas-offinder/new).
